# Histone Deacetylase 2 (HDAC2) Regulates Chromosome Segregation and Kinetochore Function via H4K16 Deacetylation during Oocyte Maturation in Mouse

**DOI:** 10.1371/journal.pgen.1003377

**Published:** 2013-03-14

**Authors:** Pengpeng Ma, Richard M. Schultz

**Affiliations:** Department of Biology, University of Pennsylvania, Philadelphia, Pennsylvania, United States of America; Baylor College of Medicine, United States of America

## Abstract

Changes in histone acetylation occur during oocyte development and maturation, but the role of specific histone deacetylases in these processes is poorly defined. We report here that mice harboring *Hdac1* ^−/+^/*Hdac2* ^−/−^ or *Hdac2* ^−/−^ oocytes are infertile or sub-fertile, respectively. Depleting maternal HDAC2 results in hyperacetylation of H4K16 as determined by immunocytochemistry—normal deacetylation of other lysine residues of histone H3 or H4 is observed—and defective chromosome condensation and segregation during oocyte maturation occurs in a sub-population of oocytes. The resulting increased incidence of aneuploidy likely accounts for the observed sub-fertility of mice harboring *Hdac2* ^−/−^ oocytes. The infertility of mice harboring *Hdac1* ^−/+^/*Hdac2* ^−/−^oocytes is attributed to failure of those few eggs that properly mature to metaphase II to initiate DNA replication following fertilization. The increased amount of acetylated H4K16 likely impairs kinetochore function in oocytes lacking HDAC2 because kinetochores in mutant oocytes are less able to form cold-stable microtubule attachments and less CENP-A is located at the centromere. These results implicate HDAC2 as the major HDAC that regulates global histone acetylation during oocyte development and, furthermore, suggest HDAC2 is largely responsible for the deacetylation of H4K16 during maturation. In addition, the results provide additional support that histone deacetylation that occurs during oocyte maturation is critical for proper chromosome segregation.

## Author Summary

Oocyte development is becoming of increasing interest not only in the broad research community but also within the general public due, in part, to the ever increasing demand for and use of assisted reproductive technologies (ART) to treat human infertility, and because the oocyte-to-embryo transition encompasses a natural reprogramming of gene expression, a process central to forming iPS cells. Dramatic changes in chromatin structure and gene expression occur during oocyte development, but the role of such changes in generating oocytes that are capable of maturing, being fertilized, and giving rise to offspring is very poorly understood. Histone deacetylases (HDACs) are critically involved in modulating chromatin structure. Here, we describe the effect of specifically deleting the gene encoding *Hdac2* in mouse oocytes and find the fertility of female mice harboring such oocytes is compromised. Although such mutant oocytes can grow they fail to mature properly to become an egg. The primary defect is that histone H4 acetylated on lysine 16 fails to become deacetylated as the oocyte matures to become an egg, with the consequence that the ability of chromosomes to interact with spindle microtubules is compromised, which in turn leads to improper chromosome segregation.

## Introduction

Post-translational modifications of histones, e.g., phosphorylation, methylation, ubiquitination, and acetylation, are critically involved in a number of cellular processes that range from regulating gene expression to repair of DNA damage [1]–[3]. Lysine acetylation of histones is controlled by histone acetyl transferases (HATs) and histone deacetylases (HDACs). In mammals, eighteen HDACs have been identified and grouped into four classes [4]. Class I enzymes HDAC1 and HDAC2 are highly homologous and ubiquitously expressed in different tissues [5]. HDAC1 and HDAC2 lack a DNA binding domain, as do all histone deacetylases, and execute their function by interacting with transcription factors as either homo- or heterodimers, or being part of multi-component repressor complexes [5].

Loss-of-function studies in mice have generated important insights regarding the function of HDAC1 and HDAC2 in regulating cell proliferation, apoptosis, and differentiation. One common theme of several tissue specific HDAC1/2 knockout studies is redundancy and compensation [6]–[9]. Nevertheless, results of other studies support the notion that HDAC1 and HDAC2 have distinct functions in some cells and tissues [10]–[13]. Taken together, these results indicate that the physiological functions of HDAC1 and HDAC2 are complicated and diversified in different tissues or cell types. Recently, we demonstrated compensatory functions of HDAC1 and HDAC2 during mouse oocyte development in which *Hdac1* and *Hdac2* were specifically deleted in oocytes [14]; deletion of both genes in oocytes results in infertility due to failure of follicle development beyond the secondary follicle stage with ensuing oocyte apoptosis attributed to hyperaceytlation of TRP53.

We also noted in that study that deleting *Hdac1* ^−/−^ in oocytes has no effect on fertility, as also observed for mice harboring *Hdac1* ^−/−^/*Hdac2* ^−/+^ oocytes. In contrast, mice in which *Hdac2* ^−/−^ was only deleted in oocytes are sub-fertile despite increased amounts of HDAC1 protein whereas their *Hdac1* ^−/+^/*Hdac2* ^−/−^ counterparts are infertile [14]. Taken together, these results suggest a more prominent role for HDAC2 than HDAC1 in oocyte development, whereas HDAC1 plays a more prominent role in preimplantation development [13]. The molecular basis for the sub-fertility of mice harboring *Hdac2* ^−/−^ oocytes or infertility of mice harboring *Hdac1* ^−/+^/*Hdac2* ^−/−^, however, was not examined.

We report here a characterization of the infertility and sub-fertilty phenotypes observed in mice harboring *Hdac1* ^−/+^/*Hdac2* ^−/−^ or *Hdac2* ^−/−^ oocytes, respectively. We find that full-grown or nearly full-grown oocytes can be obtained from mice containing *Hdac2* ^−/−^ or *Hdac1* ^−/+^/*Hdac2* ^−/−^ oocytes, respectively, albeit highly reduced numbers of *Hdac1* ^−/+^/*Hdac2* ^−/−^ oocytes are recovered. Oocytes derived from *Hdac1* ^−/+^/*Hdac2* ^−/−^ mice are not capable of supporting development due to defects beyond failure to develop past the secondary follicle stage that include abnormal spindle assembly or inability to initiate DNA replication following maturation and insemination. Similar to double mutant *Hdac1:2* ^−/−^ oocytes, *Hdac1* ^−/+^/*Hdac2* ^−/−^ oocytes obtained from secondary follicles display reduced levels of transcription and decreased amounts of histone H3K4me1-3. A subset of *Hdac1* ^−/+^/*Hdac2* ^−/−^ and *Hdac2* ^−/−^ oocytes not only fails to undergo the maturation-associated deacetylation of histone H4K16 but also fails to segregate chromosomes correctly. This failure is likely a consequence of compromised kinetochore function in mutant oocytes manifested by a decreased ability to form cold-stable microtubule-kinetochore interactions and a reduced amount of CENP-A localized to centromeres. Last, although zygotes derived from *Hdac2* ^−/−^ eggs replicate their DNA, zygotes derived from *Hdac1* ^−/+^/*Hdac2* ^−/−^ eggs do not.

## Results

### Oocyte Development Is Not as Severely Compromised in *Hdac1^−/+^/Hdac2^−/−^* Mice as Compared to *Hdac1:2^−/−^* Mice

**Figure 1:**
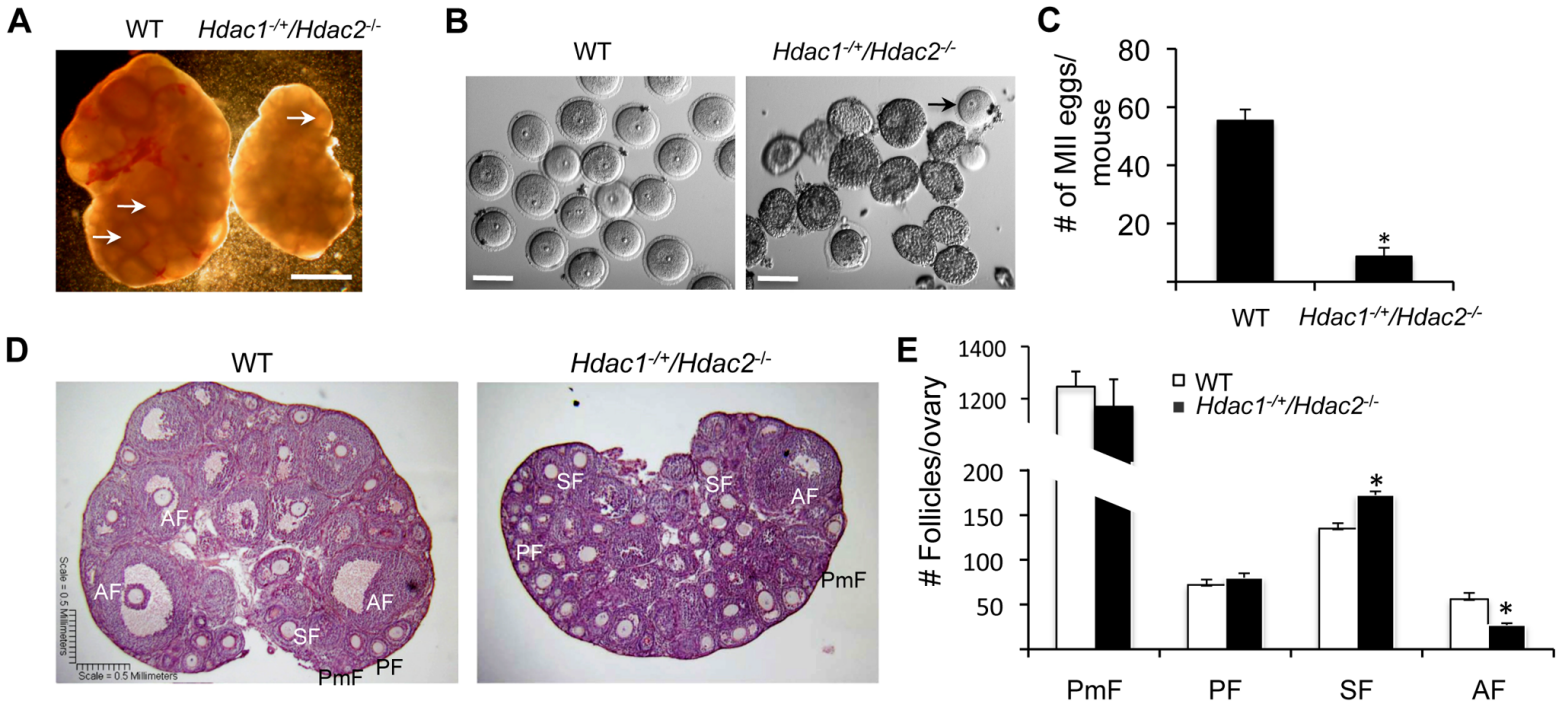
Decreased ovary size and defective oogenesis following specific targeting of *Hdac2* and reduction of *Hdac1* in oocytes. (A) Ovary morphology from WT and *Hdac1^−/+^/Hdac2* ^−/−^ mice 6 weeks-of-age. WT ovary shows presence of mature follicles (arrows), whereas fewer of such follicles are present in *Hdac1^−/+^/Hdac2* ^−/−^ mice. The bar corresponds to 1 mm. (B) Full-grown oocytes are recovered from WT mice, whereas increased number of immature follicles and only a few oocytes are recovered from *Hdac1^−/+^/Hdac2* ^−/−^ mice. The bar corresponds to 100 µm. The arrow points to an oocyte that is nearly full-grown. (C) Fewer eggs are ovulated from *Hdac1^−/+^/Hdac2* ^−/−^ mice after hormonal stimulation. WT and *Hdac1^−/+^/Hdac2* ^−/−^ 6-week-old females were superovulated with an intraperitoneal injection of 5 I.U eCG followed by administration of 5 I.U. hCG 48 h later. At least 10 mice from each genotype were used, and the average number of ovulated oocytes per female is indicated. * P<0.05. (D) Histological analysis of ovaries obtained from WT and *Hdac1^−/+^/Hdac2* ^−/−^ mice18 days-of-age. Primordial (PmF), primary (PF), secondary (SF), and antral follicles (AF) are indicated. The bar corresponds to 0.5 mm. (E) Follicle counts from ovaries obtained from WT and *Hdac1^−/+^/Hdac2* ^−/−^ mice 18 days-of-age. Data are from 3 different ovaries and are presented as mean ± SEM. * P<0.05.

We previously found that ovarian weight in 6-week-old-mice was reduced by ∼60% in *Hdac1* ^−/+^/*Hdac2* ^−/−^ mice and by ∼70% in *Hdac1:2* ^−/−^ mice compared to wild-type (WT) mice [14]. The size of ovaries obtained from 6-week old *Hdac1* ^−/+^/*Hdac2* ^−/−^ mice was intermediate of WT and double mutant mice (Figure 1A and Fig. 2 in [14]). In addition, although antral follicles were found in ovaries from *Hdac1* ^−/+^/*Hdac2* ^−/−^ mice, their number appeared decreased when compared to WT ovaries (Figure 1A); no antral follicles were found in ovaries from *Hdac1:2* ^−/−^ mice [14]. Consistent with fewer antral follicles in *Hdac1* ^−/+^/*Hdac2* ^−/−^ mice significantly fewer oocytes were obtained (Figure 1B) and fewer MII eggs were recovered following ovulation (Figure 1C). The diameter of the *Hdac1* ^−/+^/*Hdac2* ^−/−^ oocytes was about 90% that of full-grown wild-type oocytes (77.4±0.8 µm, wild-type; 69.9±1.1 µm mutant, mean ± SEM, p<0.0001).

**Figure 2:**
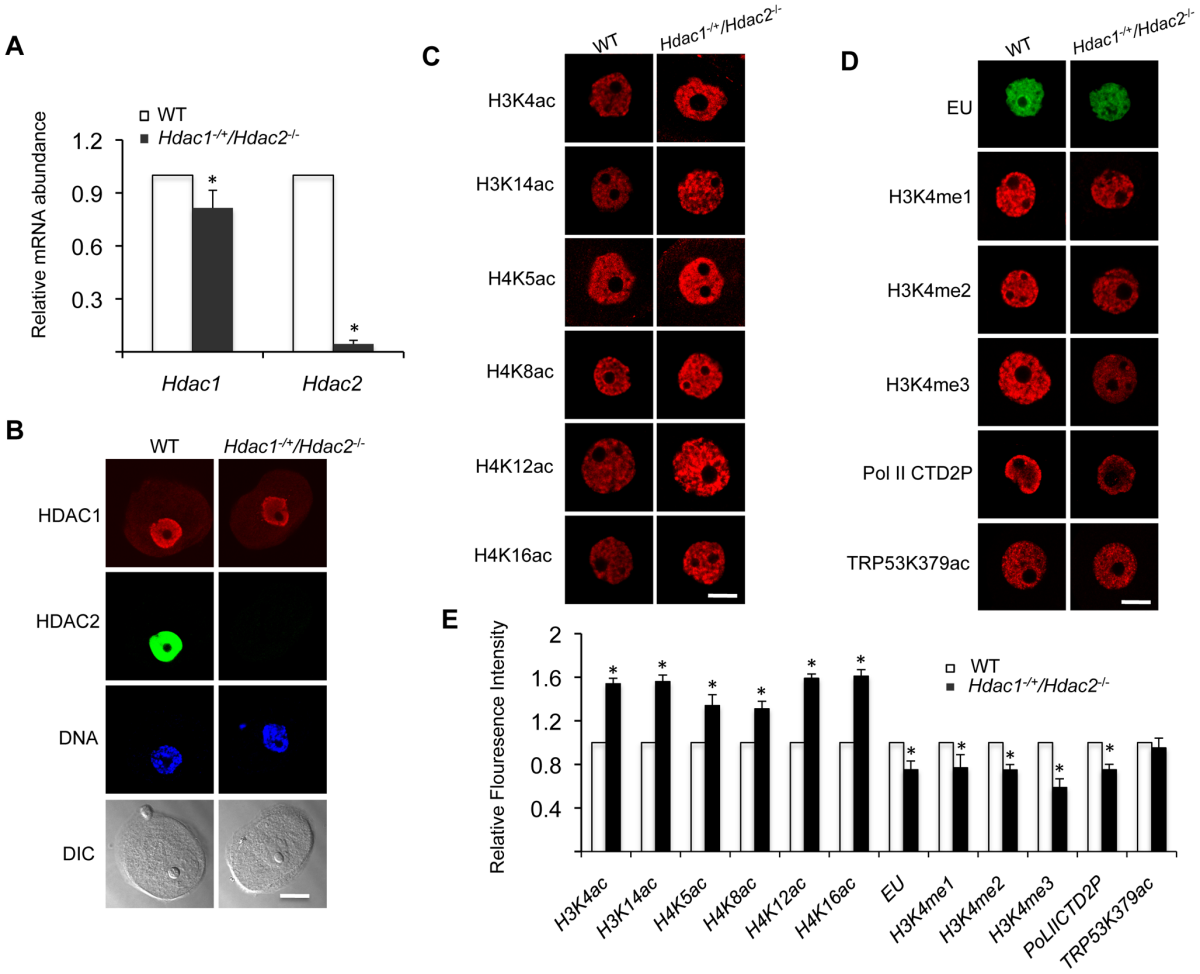
Increased histone acetylation, and decreased global transcription and histone H3K4 methylation in *Hdac1^−/+^/Hdac2^−/−^* growing oocytes. (A) Relative abundance of *Hdac1* and *Hdac2* transcripts in oocytes obtained from WT and *Hdac1^−/+^/Hdac2^−/−^* mice 12 days-of-age. Data are expressed relative to that in WT oocytes. The experiment was performed four times and the data expressed as mean ± SEM. *, P<0.05. (B) Immunocytochemical detection of HDAC1 and HDAC2 in WT and *Hdac1^−/+^/Hdac2* ^−/−^ oocytes obtained from mice 12-days-of-age. Oocytes were stained with an anti-HDAC1 antibody (Red), anti-HDAC2 antibody (Green) and DAPI (to detect DNA, Blue). Transmitted light micrographs are also shown (DIC). The bar corresponds to 20 µm. (C) Different acetylated histones were analyzed by immunocytochemistry using oocytes obtained from WT and *Hdac1^−/+^/Hdac2* ^−/−^ mice 12 days-of-age. For each histone variant, at least 20 oocytes for each genotype were analyzed, and the experiment was conducted 3 times. Shown are representative images and only the nucleus is shown. The bar corresponds to 10 µm. (D) Immunocytochemical detection of H3K4 methylation, PolII-CTD-2P, EU and TRP53K379 acethylation in WT and *Hdac1^−/+^/Hdac2* ^−/−^ oocytes obtained from mice 12-days-of-age. Shown are representative images and only the nucleus is shown. The bar corresponds to 10 µm. (E) Quantification of the data shown in panel D. Nuclear staining intensity of different proteins in WT oocytes was set to 1 and the data are expressed as mean ± SEM. At least 20 oocytes for each genotype and for each detected protein were analyzed; the experiment was conducted three times. *, p<0.05.

Oocyte development is accompanied by a change in chromatin configuration, the so-called non-surrounded nucleolus (NSN) to surrounded nucleolus (SN) configuration, which is associated with acquisition of developmental competence [15]. This transition was modestly inhibited in mutant oocytes; whereas in WT oocytes about 75% of the oocytes are in the SN configuration, we found that 46% and 51% were in the SN configuration for *Hdac1* ^−/+^/*Hdac2* ^−/−^ and *Hdac2* ^−/−^ oocytes, respectively.

Histological analysis of ovarian sections was conducted using ovaries from mice 18-days of age to capture the full spectrum of follicle development; the size of mutant ovaries was smaller than ovaries present in wild-type mice (4.25±0.12 mg vs 2.46±0.18, n = 5, p = 0.005). The analysis revealed that primary, secondary, and antral follicles were observed in *Hdac1* ^−/+^/*Hdac2* ^−/−^ ovaries (Figure 1D). Although there were no differences in the number of primordial and primary follicles, mutant ovaries had more secondary follicles but fewer antral follicles than WT ovaries (Figure 1E). Thus, development of secondary follicles to antral follicles in mutant mice appeared either delayed or inhibited, with the decrease in the number of antral follicles being similar to the increase in the number of secondary follicles. These results demonstrated that in *Hdac1* ^−/+^/*Hdac2* ^−/−^ mice oocyte development was mainly blocked at the secondary follicle stage, and one allele of *Hdac1* was not sufficient to overcome this block, supporting our previous conclusion that HDAC2 is the major HDAC in oocyte development [14]. Last, we observed no overt sign of oocyte degeneration in *Hdac1* ^−/+^/*Hdac2* ^−/−^ mice, which is in striking contrast to the increased incidence of oocyte degeneration in *Hdac1:2* ^−/−^ oocytes [14].

### Reduced Transcription in *Hdac1^−/+^/Hdac2^−/−^* Growing Oocytes Is Accompanied by Histone H3K4me1-3 Demethylation but Not Apoptosis

Our previous study characterizing the phenotype of *Hdac1:2* ^−/−^ oocytes was performed with 12-day-old mice [14]. Accordingly, we conducted studies regarding transcription and histone modifications using *Hdac1* ^−/+^/*Hdac2* ^−/−^ oocytes collected on day-12 post-partum to make valid comparisons. *Hdac2* transcripts were reduced by >95% in oocytes obtained from 12-day-old *Hdac1* ^−/+^/*Hdac2* ^−/−^ mice, whereas *Hdac1* mRNA level only decreased ∼15% (Figure 2A), which was reflected by a dramatic decrease in the nuclear staining of HDAC2 and only modest decrease (<15%) in HDAC1 nuclear staining (Figure 2B). The small decrease in *Hdac1* mRNA likely reflects a compensatory increase in *Hdac1* expression in face of loss of *Hdac2* [14]. Similar to double mutant oocytes [14], there was an increase in acetylation of both histone H3 and H4 on specific lysine residues (Figure 2C and 2E).

**Figure 3:**
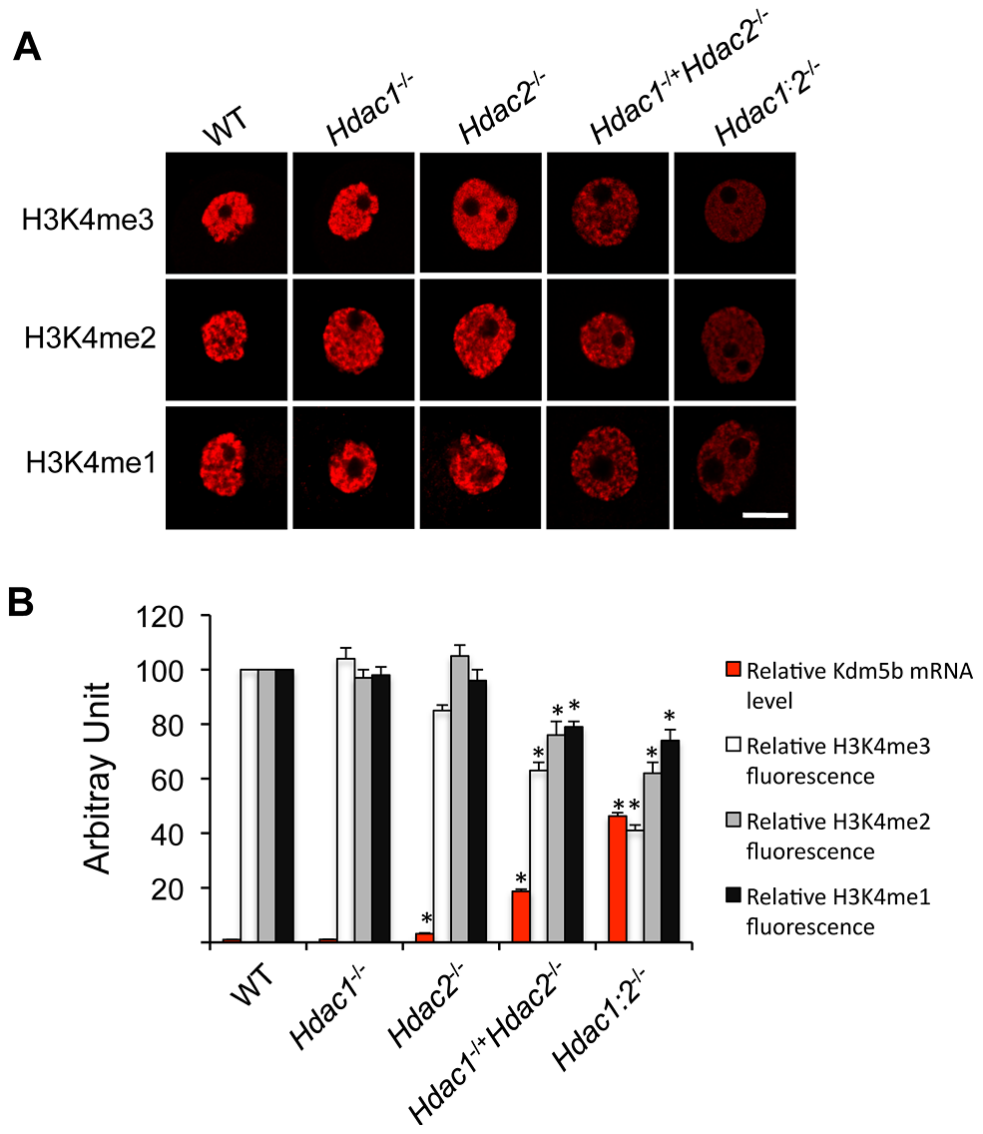
Loss of HDAC1/2 leads to H3K4me1-3 demethylation and up-regulation of KDM5B. (A) Immunocytochemical detection of H3K4me1, H3K4me2 and H3K4me3 in oocytes obtained from mice 12-days-of-age and lacking different combinations of *Hdac1* and *Hdac2*. For each histone variant, at least 20 oocytes for each genotype were analyzed, and the experiment was conducted 3 times. Shown are representative images and only the nucleus is shown. The bar corresponds to 10 µm. (B) Quantification of the data shown in panel A and relative abundance of *Kdm5b* mRNA in different genotype oocytes obtained from mice 12-days-of-age. For immunoflurosecence quantification, the nuclear staining intensity of H3K4me1-3 in the WT oocytes was set to 100. The relative abundance of *Kdm5b* transcript was assayed by qRT-PCR and expressed relative to WT *Kdm5b* mRNA level that was set as 1. UBF was used as internal control. All data are expressed as mean ± SEM.

Transcription was reduced by ∼20%, as assayed by EU incorporation, in *Hdac1* ^−/+^/*Hdac2* ^−/−^ oocytes when compared to WT oocytes (Figure 2D and 2E), being intermediate when compared to *Hdac1:2* ^−/−^ oocytes in which transcription is reduced by 40% [14]. *Hdac2* ^−/−^ oocytes did not exhibit any significant decrease in the extent of EU incorporation relative to WT (53±4 relative units vs 48±2 relative units, respectively; p>0.20. The data are expressed as mean ± SEM, and the number of oocytes examined was 16 and 24, respectively). We also observed a significant decrease in the intensity of H3K4me1&2 (∼20%) and H3K4me3 (∼40%) nuclear staining in *Hdac1* ^−/+^/*Hdac2* ^−/−^ oocytes (Figure 2D and 2E); active promoters are marked in general by trimethylated H3K4 (H3K4me3), whereas dimethylated H3K4 (H3K4me2) is often found in the coding region [16], [17]. A decrease in H3K4me1 of ∼20%, and a decrease in H3K4me2 and H3K4me3 of ∼35% and 60%, respectively were observed in *Hdac1:2* ^−/−^ oocytes (Figure 3 and Fig. 6D in [14]). By using antibodies that specifically recognize CTD S2 phosphorylation, which is a marker for RNA polymerase II engaged in transcription [18], a significant reduction (∼20%) was observed in CTD S2 phosphorylation in *Hdac1* ^−/+^/*Hdac2* ^−/−^ growing oocytes (Figure 2D and 2E). This decrease was consistent with the 20% decrease in global transcription.

We previously observed a 40-fold increase in the amount of *Kdm5b* transcript in *Hdac1:2* ^−/−^ oocytes and no change in *Hdac1* ^−/−^ oocytes [14]; KDM5B is apparently the only histone lysine demethylase that can demethylate H3K4me3, H3K4me2 and H3K4me1 [19]. *Kdm5b* transcripts were also increased, as determined by qRT-PCR, by 18-fold in *Hdac1* ^−/+^/*Hdac2* ^−/−^ oocytes and 3-fold in *Hdac2* ^−/−^ oocytes. The extent of up-regulation of *Kdm5b* transcripts was related to the extent of loss of *Hdac1* and *Hdac2*, with a corresponding decrease in histone H3K4me1-3 (Figure 3B). These results suggest that KDM5B plays a significant role in establishing the steady-state amount of methylated histone H3K4. In contrast to the dramatic increase in TRP53K379 acetylation that occurs in *Hdac1:2* ^−/−^ oocytes [14] and results in increased TRP53 activity [20], [21], TRP53 was not hyperacetylated in *Hdac1* ^−/+^/*Hdac2* ^−/−^ oocytes (Figure 2D and 2E), consistent with apoptosis not being observed in oocytes from these mice. The absence of TRP53 acetylation suggests that only one allele of *Hdac1* or *Hdac2* is sufficient to prevent increased TRP53 activity in growing oocytes.

### Depletion of Maternal HDAC2 Leads to Hyperacetylation of H4K16 and Defective Chromosome Condensation and Segregation during Oocyte Maturation

Although most *Hdac1^−/+^/Hdac2^−/−^* oocytes were arrested within secondary follicles (Figure 1D and 1E), a small number of nearly full-grown oocytes could be recovered from *Hdac1^−/+^/Hdac2^−/−^* ovaries (Figure 1B). A single mutant of *Hdac1* or *Hdac2* has no apparent effect on oocyte maturation and subsequent preimplantation development [14] and Figures S1 and S2). We next asked if depletion of HDAC2 combined with a reduction of HDAC1 has any effect on oocyte maturation by using *Hdac^−/+^/Hdac2^−/−^* oocytes.

**Figure 4:**
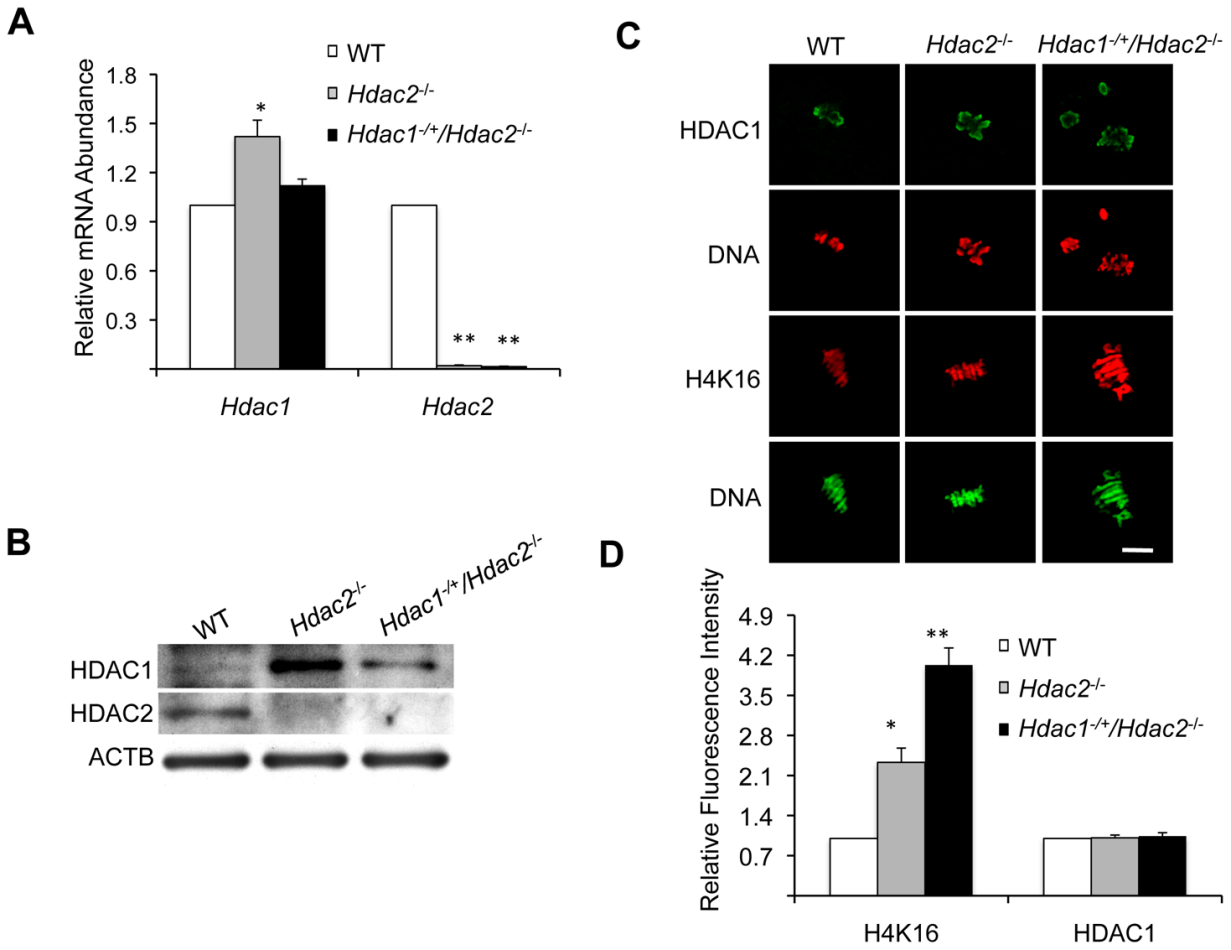
Depletion of maternal HDAC2 results in increased histone H4K16 acetylation following oocyte maturation. (A) Relative abundance of *Hdac1* and *Hdac2* transcripts in full-grown oocytes obtained from WT, *Hdac2^−/−^* and *Hdac1^−/+^/Hdac2^−/−^* mice. Data are expressed relative to that in WT oocytes. The experiment was performed four times and the data expressed as mean ± SEM. *, p<0.05; **, p<0.001. (B) Immunoblot analysis of HDAC1 and HDAC2 expression in WT and mutant full-grown oocytes; total protein was extracted from fully-grown oocytes (180 for HDAC1 and 80 for HDAC2) obtained from WT, *Hdac2^−/−^* and *Hdac1^−/+^/Hdac2^−/−^* mice for immunoblotting. The experiment was conducted 3 times, and similar results were obtained in each case. ACTB was used as a loading control. (C) Immunocytochemical detection of HDAC1 and histone H4K16 acetylation in WT, *Hdac2^−/−^* and *Hdac1^−/+^/Hdac2* ^−/−^ MII eggs; at least 20 oocytes from each genotype were analyzed, and the experiment was conducted 3 times. Shown are representative images and the DNA was stained with Sytox Green (green) or propidium iodide (Red). The bar corresponds to 10 µm. (D) Quantification of the data shown in panel C. Staining intensity of different proteins in WT eggs was set to 1 and the data are expressed as mean ± SEM. At least 20 eggs for each genotype and for each detected protein were analyzed; the experiment was conducted three times. *, p<0.05; **, p<0.001.

We first checked *Hdac1* and *Hdac2* expression in full-grown *Hdac1^−/+^/Hdac2^−/−^* oocytes. qRT-PCR revealed that *Hdac2* mRNA was reduced by >98% in *Hdac1^+/−^/Hdac2^−/−^* full-grown oocytes (Figure 4A). *Hdac1* mRNA levels, however, showed no difference between *Hdac1^−/+^/Hdac2^−/−^* and WT oocytes (Figure 4A), which suggests an ∼2-fold compensatory increase in *Hdac1* expression in *Hdac1^−/+^/Hdac2^−/−^* oocytes. Consistent with these results, the amount of HDAC1 protein exhibited a mild increase in *Hdac1* ^−/+^ */Hdac2^−/−^* oocytes when compared to WT oocytes (Figure 4B), whereas there was a significant increase of both *Hdac1* mRNA and HDAC1 protein in *Hdac2^−/−^* oocytes (Figure 4A and 4B). Because the amount of HDAC1 present in *Hdac1^−/+^/Hdac2^−/−^* and *Hdac2* ^−/−^ oocytes was similar to or more than that in WT oocytes, *Hdac1^−/+^/Hdac2^−/−^* and *Hdac2* ^−/−^ oocytes provide a system to assess the role of maternal HDAC2 in oocyte maturation and subsequent fertilization and preimplantation development.

Genome-wide histone deacetylation mediated by HDACs at several lysine residues occurs during oocyte maturation in mouse [22], [23], [24] and pig [25]. The identity of the responsible HDACs, however, is poorly defined in mouse. Accordingly, we first assessed the effect of deleting maternal HDAC2 on histone acetylation during oocyte maturation. Remarkably, immunostaining revealed that although the amount of HDAC1 associated with chromosomes was unchanged in mutant oocytes, the acetylation state of histone H4K16 was affected, being increased ∼2.3-fold in *Hdac2^−/−^* and ∼4-fold in *Hdac1^−/+^/Hdac2^−/−^* MII eggs (Figure 4C and 4D); the maturation-associated deacetylation of histone H4K5, H4K8, H4K12, H3K4, H3K9, and H3K14 appeared to occur normally in these cells (Figure S3). It was unlikely that an increase in MYST1, an H4K16-specific histone acetyltransferase [26] and largely responsible for H4K16 acetylation [27], accounted for the observed increase in acetylated H4K16 following maturation because there was no obvious change in the amount of MYST1 protein in *Hdac1^−/+^/Hdac2^−/−^* or *Hdac2* ^−/−^ oocytes (Figure S4A). The amount of acetylated H416 was increased in growing *Hdac1^−/+^/Hdac2^−/−^* oocytes, suggesting nuclear HDAC2 can deacetylate H4K16 (Figure 2C and 2D). Nuclear HDAC2 in full-grown oocytes, however, appeared unable to deacetylate H4K16 because similar amounts of acetylated H4K16 were observed in full-grown WT and *Hdac1^−/+^/Hdac2^−/−^* oocytes (Figure S4B).

**Figure 5:**
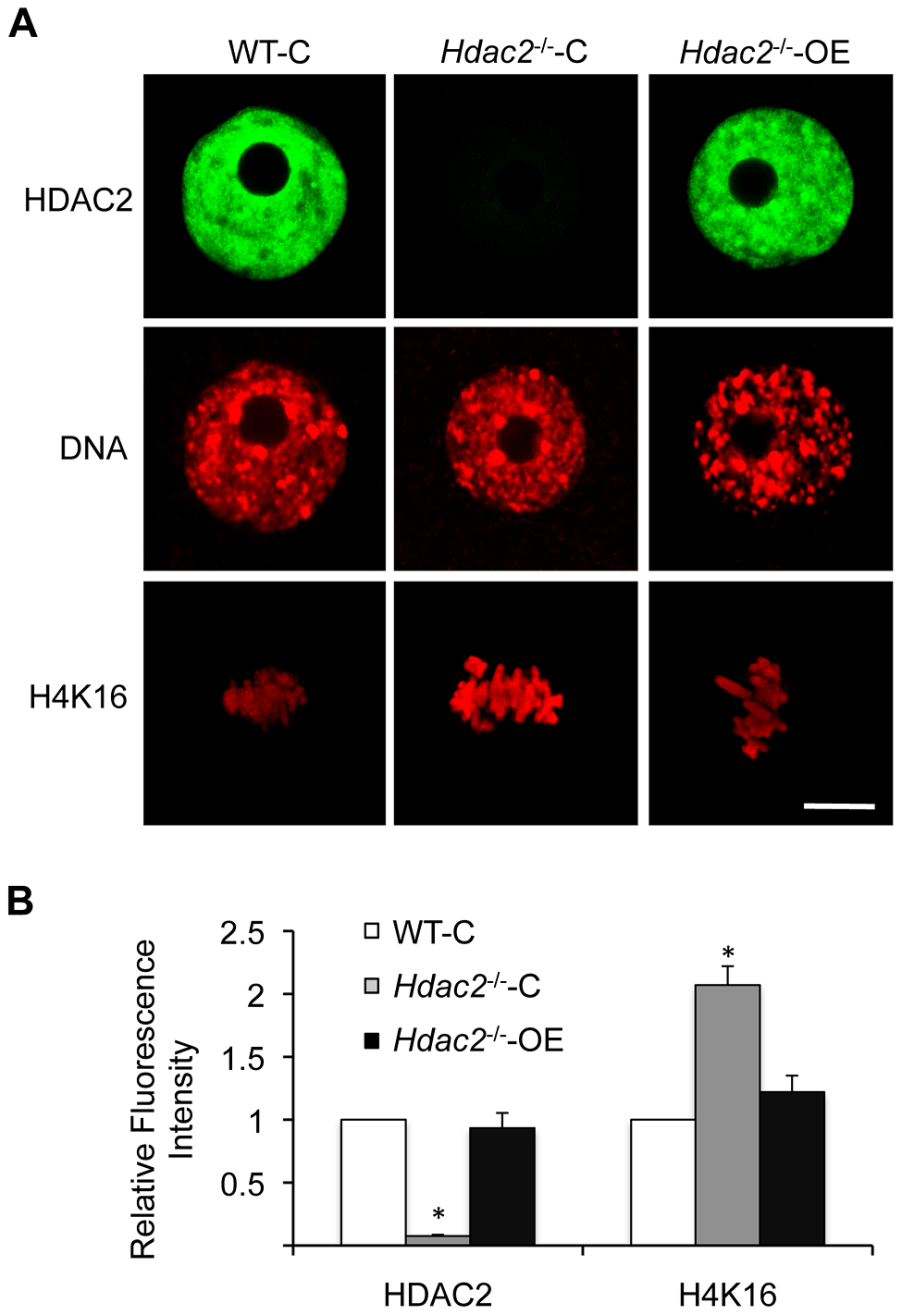
Expression of HDAC2 in *Hdac2* ^−/−^ oocytes restores maturation-associated deacetylation of histone H4K16. (A) Mutant oocytes were injected with a cRNA (0.4 µg/µl) encoding either *Egfp* (*Hdac2^−/−^-*C) or *Hdac2* (*Hdac2^−/−^-*OE) and incubated 24 h in CZB containing milrinone to prevent maturation; controls were wild-type oocytes (WT-C). A portion of the cells was removed for immunoctyochemical detection of HDAC2 whereas the other portion was allowed to mature following transfer to milrinone-free medium. The eggs were then processed for immunocytochemical detection of acetylated histone H4K16. At least 8 cells were analyzed in each group. Shown are representative images. DNA was counterstained with propidium iodide. The bars corresponds to 10 µm. (B) Quantification of the relative amount of acetylated histone H4K16. The data are expressed as mean ± SEM. *, p<0.01.

The increased acetylation of H4K16 following maturation of mutant oocytes was presumably due to loss of HDAC2 activity because *Hdac2* ^−/−^ oocytes injected with an *Hdac2* cRNA, but not an *Egfp* cRNA, exhibited the maturation-associated decrease in acetylated H4K16 to a similar degree as observed in wild-type oocytes (Figure 5). Note that HDAC2 was expressed to similar levels in mutant oocytes when compared to wild-type oocytes, minimizing the likelihood that deacetylation of H4K16 was due to off-targeting effects. In addition, chromosomes appeared less condensed in *Hdac1^−/+^/Hdac2^−/−^* and *Hdac2* ^−/−^ MII eggs; whereas only 2.4% of WT eggs (4 out of 163) had chromosomes that appeared not fully condensed, this incidence increased to 30.8% (28 out of 91) and 44.9% (40 out of 89) in *Hdac2* ^−/−^ and *Hdac1^−/+^/Hdac2^−/−^* MII eggs, respectively.

**Figure 6:**
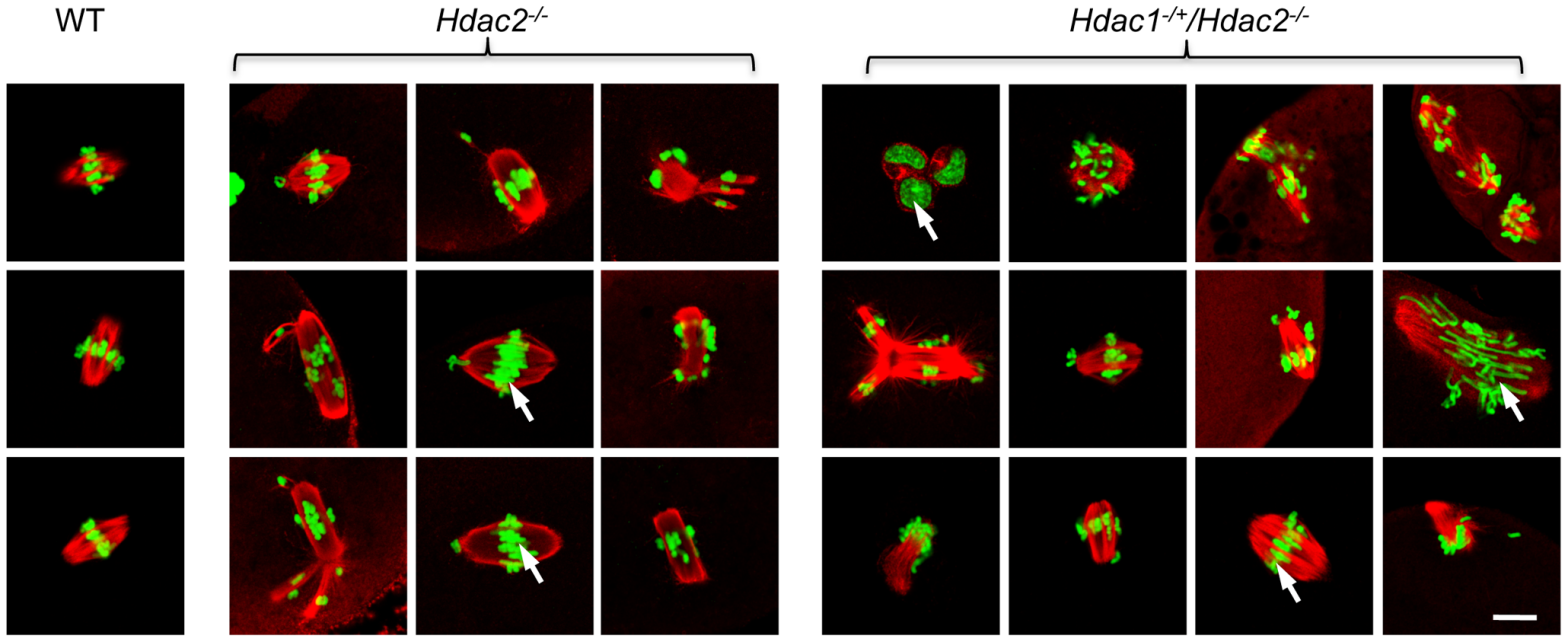
Loss of maternal HDAC2 causes defective chromosome condensation and congression in MII eggs. Spindle morphology in WT, *Hdac2^−/−^* and *Hdac1^−/+^/Hdac2^−/−^* MII eggs. MII eggs were fixed and stained with anti-TUBB antibody (red); DNA was counterstained with Sytox green. Representative images are shown. The bar corresponds to 10 µm.

**Figure 7:**
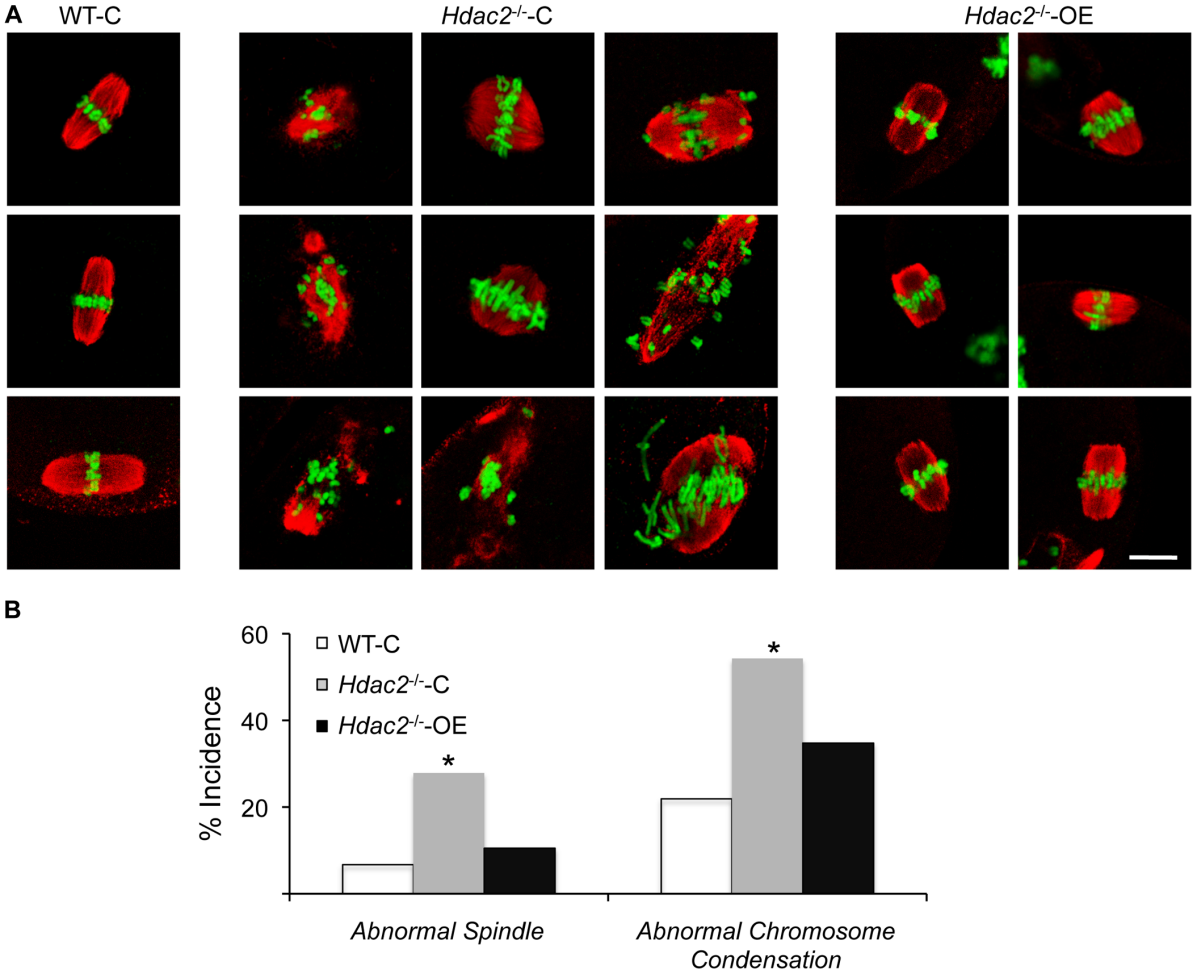
Expression of HDAC2 in *Hdac2^−/−^* oocytes restores normal chromosome condensation and spindle formation in MII eggs. (A) Spindle morphology and chromosome alignment in MII eggs. Mutant oocytes were injected with a cRNA (0.4 µg/µl) encoding either *Egfp* (*Hdac2^−/−^-*C) or *Hdac2* (*Hdac2^−/−^-*OE) and incubated 24 h in CZB containing milrinone to prevent maturation; controls were wild-type oocytes injected with *Egfp* cRNA (WT- C). The oocytes were allowed to mature following transfer to milrinone-free medium overnight. MII eggs were fixed and stained with ß-tubulin antibody (red); DNA was counterstained with Sytox green (green). Shown are representative images. The bar corresponds to 10 µm. (B) Frequency of abnormal spindles and abnormal chromosome condensation in WT-C, *Hdac2^−/−^-*C and *Hdac2^−/−^-*OE MII eggs. 104 WT-C eggs, 104 *Hdac2^−/−^-*C eggs and 76 *Hdac2^−/−^-*OE eggs were analyzed respectively. *, p<0.05, χ^2^.

The effect of deleting *Hdac1* and *Hdac2* on the acetylation state of H4K16 and chromosome condensation next led us to examine spindle formation and chromosome alignment in *Hdac1^−/+^/Hdac2^−/−^* and *Hdac2^−/−^* eggs following oocyte maturation. Analysis of meiotic spindle configurations in MII eggs revealed a significant increase in the proportion of eggs with abnormal spindles and misaligned chromosome in *Hdac2^−/−^* and *Hdac1^−/+^/Hdac2^−/−^* eggs when compared to WT eggs (Table 1 and Figure 6). As anticipated the incidence of aneuploidy was increased in *Hdac1^−/+^/Hdac2^−/−^* and *Hdac2^−/−^* eggs (Table 2 and Figure S5). Absence of HDAC2 protein was likely the proximate cause for the observed increased incidence of abnormal spindles and misaligned chromosomes because over-expressing HDAC2 in *Hdac2* ^−/−^ oocytes restored, in large part, the WT phenotype (Figure 7).

**Table 1:** Incidence of abnormal spindles in *Hdac* mutant MII eggs.

	WT	*Hdac2* ^−/−^	*Hdac1* ^−/+^/*Hdac2* ^−/−^
# MII eggs with abnormal spindle	1	12	21
# Total MII eggs analyzed	125	148	135
% Abnormal MII	0.8	8.8*	15.6**

Difference between WT and mutant is significant, *P<0.05, **P<0.01, χ^2^. The difference between *Hdac2^−/−^*and *Hdac1^−/+^Hdac2^−/−^* MII eggs is also significant, P<0.05.

**Table 2:** Incidence of aneuploidy in *Hdac1^−/+^/Hdac2^−/−^* and *Hdac2^−/−^* eggs.

Genotype	# Aneuploid	Total #	% Aneuploid
WT	1	77	1.3
*Hdac2^−/−^*	11	90	12.2*
*Hdac1^−/+^/Hdac2^−/−^*	11	60	18*

* p<0.01. The difference between *Hdac1^−/+^/Hdac2^−/−^* and *Hdac2^−/−^* eggs is not significant.

**Figure 8:**
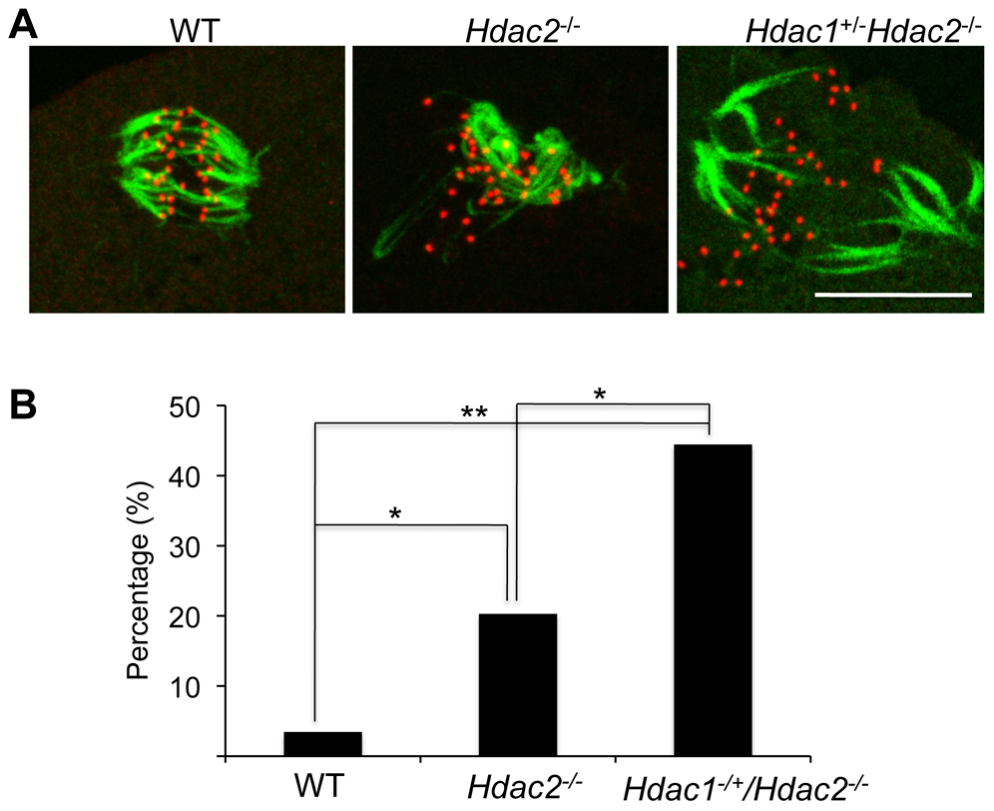
Loss of maternal HDAC2 impairs kinetochore-microtubule attachment in MI oocytes. (A) Full-grown oocytes from WT, *Hdac2^−/−^* and *Hdac1^−/+^/Hdac2^−/−^* mice were matured to MI by culturing them in CZB medium for 7 h prior to assaying for the presence of cold-stable microtubules. Kinetochores were detected by immunocytochemistry using CREST autoimmune serum (red) and anti-TUBB antibody (green) was used to detect microtubules. Shown are representative images. The bar corresponds to 10 µm. (B) Frequency of abnormal spindles and impaired kinetochore-microtubules attachment in WT, *Hdac2^−/−^*and *Hdac1^−/+^/Hdac2^−/−^* MI oocytes. 58 WT oocytes, 69 *Hdac2^−/−^* oocytes and 54 *Hdac1^−/+^/Hdac2^−/−^* oocytes were analyzed respectively. *, p<0.05, **, p<0.01, χ^2^.

**Figure 9:**
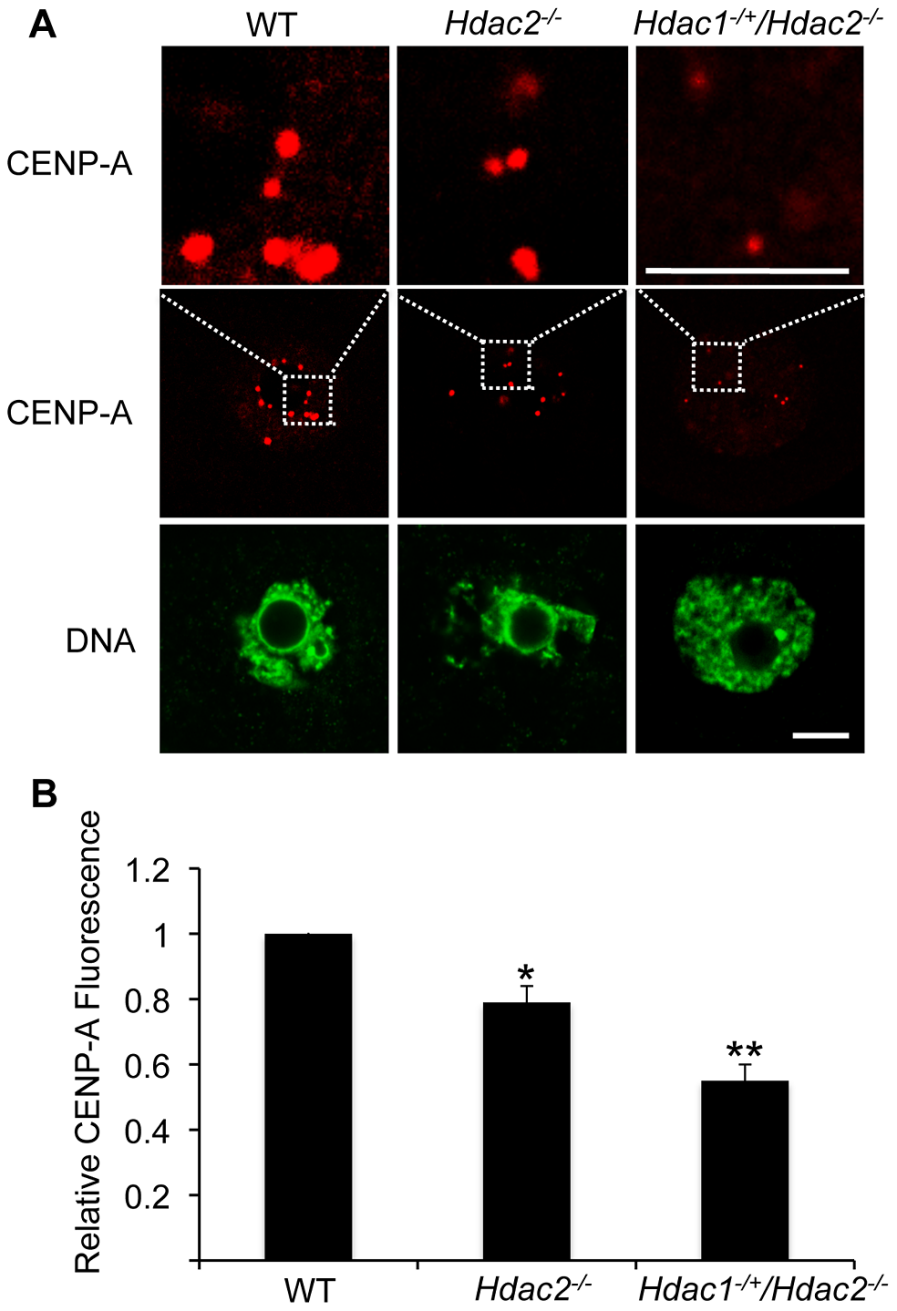
Deletion of maternal *Hdac2* leads to reduced CENP-A expression in mouse oocytes. (A) Immunocytochemical detection of CENP-A in WT, *Hdac2^−/−^* and *Hdac1^−/+^/Hdac2^−/−^* full-grown oocytes; at least 20 oocytes from each genotype were analyzed, and the experiment was conducted 2 times. Shown are representative images and DNA was counterstained with Sytox green (green). The bar corresponds to 10 µm. The upper row shows an enlargement of the region in the outlined box. (B) Quantification of the data shown in panel A. Staining intensity of different proteins in WT eggs was set to 1 and the data are expressed as mean ± SEM. Signal intensities relative to WT in *Hdac2^−/−^* and *Hdac1^−/+^/Hdac2^−/−^* are 81±4%, and 55±6% respectively. *, p<0.05; **, p<0.01.

In somatic cells, histone hyperacetylation can interfere with kinetochore assembly [28] and in budding yeast, which have 125 bp “point” centromeres (which contrasts to 0.1–5 Mb bp “regional” centromeres in fission yeast and humans), hypoacetylation of H4K16 is critical to maintain kinetochore function [29]. Hyperacetylation of H4K16 in oocyte lacking HDAC2, therefore, could also compromise kinetochore function and lead to the increased incidence of misaligned chromosomes on the spindle. Such appears to be the case. Most spindle microtubules depolymerize at low temperature, except for kinetochore microtubules, which are preferentially stabilized [30], i.e., compromised kinetochore function leads to a decrease in the number of cold-stable microtubules. Finding that the incidence of kinetochores, as detected by CREST staining, not associated with cold-stable microtubules was significantly increased in mutant oocytes strongly implies that kinetochore function is compromised (Figure 8). This conclusion is further buttressed by observing less CENP-A staining at centromeres in mutant oocytes (Figure 9).

### *Hdac1^−/+^/Hdac2^−/−^* Eggs Are Fertilized but Fail to Initiate DNA Replication

**Figure 10:**
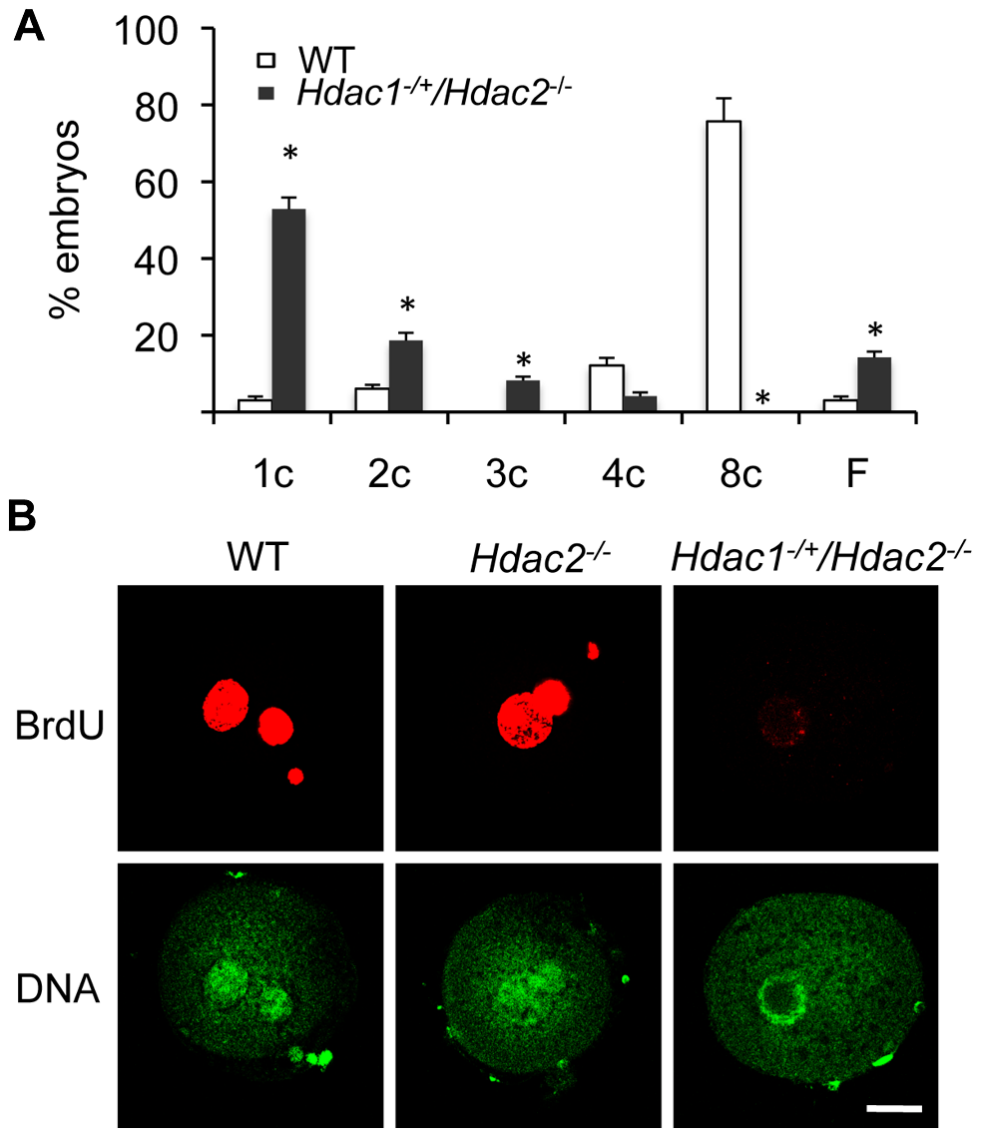
Deletion of maternal HDAC2 results in failure to replicate DNA 1-cell embryos. (A) Embryos derived from *Hdac1^−/+^/Hdac2^−/−^* females crossed to WT males arrest early in development. Results are presented as % embryos (average) ± SEM from 3 independent experiments. Abbreviations: c, cell; F, fragmented. (B) Confocal images of WT, *Hdac2^−/−^* and *Hdac1^−/+^/Hdac2^−/−^* 1-cell embryos in which the incorporation of BrdU was detected by immunocytochemistry. Shown are representative images and DNA was counterstained with Sytox green (green). The experiment was conducted twice and at least 12 embryos were analyzed for each group. Only one of the two pronuclei was in the focal plane for the *Hdac1* ^+/−^/*Hdac2* ^−/−^ sample. The bar corresponds to 10 µm.

The incidence of aneuploidy in *Hdac2^−/−^* eggs could account for the observed sub-fertility in mutant female mice, but would not account for the infertility in *Hdac1^−/+^/Hdac2^−/−^* mice. Accordingly we ascertained whether *Hdac1^−/+^/Hdac2^−/−^* eggs could be fertilized, and if so assessed their ability to develop. *Hdac1^−/+^/Hdac2^−/−^* eggs were readily fertilized as evidenced by formation of a male and female pronucleus; only small fraction (∼15%) of these eggs failed to be fertilized. When *Hdac1^−/+^/Hdac2^−/−^* female mice were mated to wild-type males, no blastocysts were recovered following development *in vivo*. Performing a similar mating in which 1-cell embryos were recovered and then permitted to develop *in vitro* demonstrated that most *Hdac1^−/+^/Hdac2^−/−^* embryos arrested at the 1-cell (53%) and 2-cell (∼20%) stages, and a higher incidence of fragmentation was observed (Figure 10A).

The high incidence of failure of *Hdac1^−/+^/Hdac2^−/−^* 1-cell embryos to cleave to the 2-cell stage led us to examine whether failure to undergo DNA replication was the cause. Whereas BrdU was readily incorporated by zygotes obtained from WT and *Hdac2^−/−^* eggs, all zygotes obtained from *Hdac1^−/+^/Hdac2^−/−^* eggs failed to incorporate BrdU (Figure 10B). The 2-cell embryos observed following insemination of *Hdac1^−/+^/Hdac2^−/−^* eggs, therefore, were likely the products of pseudo-cleavage. Last, BrdU incorporation by zygotes obtained from *Hdac2^−/−^* eggs suggests that the up-regulation of HDAC1 in *Hdac2^−/−^* oocytes compensates for loss of HDAC2.

Deleting both *Hdac1* and *Hdac2* in dividing cells results in a cell cycle block in G1 phase through induction of expression of the cyclin-dependent kinase inhibitors P21 and P57 [9]. Due to the similar phenotype observed in zygotes derived from *Hdac1^−/+^/Hdac2^−/−^* eggs, we asked whether a similar situation occurred. Although there was an increased abundance of *p57* and *p21* transcripts in these zygotes compared to WT, there was no obvious increase in the amount of P57 or P21 protein (Figure S6A and S6B).

DNA replication in 1-cell embryos appears to require recruitment of two maternal mRNAs during maturation, namely, CDC6 and ORC6L [31], [32]. *Cdc6* and *Orc6l* mRNAs were effectively recruited during maturation as evidenced by strong fluorescence signal observed in the pronuclei (Figure S6C); little or no CDC6 or ORCL protein is present in oocytes [31], [32]. This finding minimizes the likelihood that insufficient amounts of CDC6 and ORC6L protein were the cause for failure of fertilized *Hdac1^−/+^/Hdac2^−/−^* eggs to initiate DNA replication.

## Discussion

We previously demonstrated that specifically deleting in oocytes both *Hdac1* and *Hdac2* genes led to developmental failure beyond the secondary follicle stage, the likely consequence of a 40% decrease in transcription, a massive perturbation in the transcriptome, and TRP53 hyperacetylation leading to apoptosis. The results reported here extend our understanding of HDAC1 and HDAC2 functions during oocyte development by identifying processes they control during oocyte maturation and following fertilization.

The ability of *Hdac1^−/+^/Hdac2^−/−^* or *Hdac2* ^−/−^ oocytes to reach the nearly full-grown or full-grown stage within preovulatory antral follicles is likely a consequence that they fail to undergo apoptosis because TRP53 is not hyperacetylated. Nevertheless, only a small fraction of *Hdac1^−/+^/Hdac2^−/−^* oocytes develop beyond the secondary follicle stage, whereas development beyond the secondary follicle stage is quite robust in *Hdac2* ^−/−^ oocytes. This difference may reflect that global transcription is reduced by 20% in *Hdac1^−/+^/Hdac2^−/−^* oocytes but unaffected in *Hdac2^−/−^* oocytes. The perturbation in transcription in *Hdac1^−/+^/Hdac2^−/−^* oocytes could impact the communication that exists between oocytes and the surrounding granulosa/cumulus cells and is essential for both oocyte growth and follicle cell proliferation [33]. Although not determined here, the transcriptome of *Hdac1^−/+^/Hdac2^−/−^* oocytes is likely dramatically perturbed as it is in double knock out oocytes [14]. That such an alteration could compromise follicle cell function, is supported by our finding that whereas ovarian weight increases ∼2-fold following eCG priming of 21-day-old WT mice, ovaries from mutant mice fail to respond (data not shown). We also find that deleting *Hdac2* results in an increased incidence of aneuploidy that is associated with hyperacetylation of H4K16 following oocyte maturation, a finding implicating HDAC2 in chromosome condensation and segregation. Last, depletion of HDAC2 combined with reduction of HDAC1 in *Hdac1^−/+^/Hdac2^−/−^* oocytes results in a subpopulation that can mature to MII, but following egg activation, fail to replicate their DNA.

There is no apparent effect on fertility of *Hdac1^−/−^/Hdac2^−/+^* mice [14], whereas *Hdac1^−/+^/Hdac2^−/−^* mice are infertile, their infertility attributed to a combination of defects in oocyte development, maturation, and embryo development. These results provide further support that HDAC2 plays a more prominent role during oocyte development, whereas HDAC1, which is zygotically expressed, is the major HDAC regulating preimplantation development [13]. HDAC1 function during oocyte maturation and following fertilization remains less defined. The amount of HDAC1 protein in *Hdac1* ^−/−^ full-grown oocytes is ∼55% that of WT oocytes (Figure S1B), confounding analysis of HDAC1 function because such mice are fully fertile [14]. The highest levels of *Hdac1* expression are in the primordial and primary follicle stages [14], [34], which may explain why the *Zp3*-*Cre* mediated strategy did not effectively deplete HDAC1; the *Zp3* promoter becomes active during the primordial to primary follicle transition [35].

Oocyte growth is accompanied by a transition from the so-called non-surrounded nucleolus (NSN) configuration in which condensed chromatin does not surround the nucleolus to surrounded nucleolus (SN) configuration that is characterized by highly condensed chromatin around the nucleolus [36], [37]. During this transition, which is uncoupled for the onset of transcriptional quiescence [22], [38], [39], there is an increase in both histone acetylation and methylation [40]. These increases that occur during oocyte growth are not affected in *Hdac2* ^−/−^ oocytes (Figure S7A, S7B), i.e., full-grown mutant SN oocytes display the increase whereas mutant NSN oocytes do not.

*Hdac1* ^−/+^ */Hdac2^−/−^* oocytes that develop beyond the secondary follicle stage and are capable of undergoing meiotic maturation display two phenotypes. One phenotype is a failure to condense fully chromosomes (see below for further discussion of HDAC2 in chromosome dynamics). For those oocytes that mature to MII, the phenotype is a failure to undergo DNA replication following insemination. Whether the failure to replicate DNA is directly linked to altered histone acetylation or a consequence of perturbed transcription during oocyte development/acetylation of non-histone proteins is unknown. In general, however, there is a positive relationship between histone acetylation and DNA replication ([41] and references therein), possibly a consequence of increased chromatin accessibility that facilitates pre-replicative complex assembly. Last, the observed early developmental arrest is similar to several maternal-effect mutants (e.g., *Npm2, Stella, Zar1, Hsf1, MLL2*, and *Mater*) [38], [39], [42]–[45], which arrest primarily at the 1-2-cell stage. No DNA replication defects, however, were reported for these mutants, suggesting a different mechanism for developmental arrest.

Mouse oocyte maturation is accompanied by a global decrease in acetylated H3 and H4 histones [22]–[24], but the responsible HDACs have yet to be identified. Histone H3 and H4 deacetylation are not observed during M-phase for preimplantation embryos, except for deacetylation of H4K5 [23]. A similar situation is observed in NIH 3T3 cells, i.e., only H4K5 is deacetylated during M phase [23]. HDACs are clearly active in full-grown GV-intact oocytes because treatment with TSA, an HDAC inhibitor, results in increased histone acetylation [22]. Likewise, histone hyperacetylation is observed in growing oocytes in which *Hdac1* and *Hdac2* have been specifically deleted [14]. The amount of acetylated histone in GV-intact oocytes, therefore, represents a steady-state level reflecting HDAC and HAT activities. HDAC activity presumably outstrips HAT activity following maturation, thus accounting for the global decrease in histone acetylation. For example, treating MII eggs with TSA does not lead to an increase in histone acetylation, suggesting that HATs are inactive (or cannot access their histone substrates). In contrast, HDACs remain functional (or have access to their histone substrates) because oocytes matured in the presence of TSA fail to exhibit histone deacetylation but do so shortly following transfer to TSA-free medium [23].

HDAC2 is clearly implicated in the maturation-associated deacetylation of H4K16 because an increase in H4K16 is observed in *Hdac1^−/+^/Hdac2^−/−^* and *Hdac2^−/−^* MII eggs, but not in *Hdac1^−/−^/Hdac2^−/+^* oocytes (Figure S7C). The apparent greater increase in acetylated H4K16 in *Hdac1^−/+^/Hdac2^−/−^* MII eggs when compared to *Hdac2^−/−^* MII eggs (Figure 4C) also suggests a role for HDAC1 in controlling the acetylation state of H4K16, albeit less than that of HDAC2. It should be noted that HDAC2 is not associated with chromosomes in MII eggs [13], [14] and the amount of chromosome-associated HDAC1 is similar in *Hdac1^−/+^/Hdac2^−/−^* and *Hdac2^−/−^* MII eggs (Figure 4C). Because the total amount of HDAC1 is greater in *Hdac2^−/−^* oocytes than in *Hdac1^−/+^/Hdac2^−/−^* oocytes, HDAC1 not associated with chromosomes may be responsible for the decreased amount of acetylated H4K16 in *Hdac2^−/−^* eggs when compared to *Hdac1^−/+^/Hdac2^−/−^* eggs.

To date, only class III HDACs (sirtuins) have been shown to deacetylate specifically histone H4K16 [46]. Taken together, our results suggest that HDAC2 is the HDAC largely responsible for the maturation-associated deacetylation of H4K16, i.e., Class I HDACs are involved. This conclusion contrasts with the conclusion drawn from a study using porcine oocytes that Class I HDACs are not involved and was based on the inability of valproic acid, an inhibitor of Class I HDACs, to inhibit the maturation-associated decrease in histone acetylation [47]. That study, however, only assayed for the acetylation state of histone H4K8 and H3K14, and not H4K16, which could reconcile the different conclusions. Last, other acetylated lysine residues in H3 and H4 are deacetylated during maturation in both *Hdac1^−/+^/Hdac2^−/−^* or *Hdac2* ^−/−^ oocytes. The identity of the responsible HDACs remains to be determined.

A striking finding reported here is the increase in H4K16 acetylation in virtually all oocytes lacking HDAC2 following oocyte maturation and the associated failure of chromosomes to condense fully and align on the metaphase plate properly in a subpopulation; these failures presumably underlie the observed increased incidence in aneuploidy. That not all mutant oocytes show abnormal chromosome condensation and chromosome alignment despite an increase in H4K16 acetylation in all mutant oocytes is consistent with the finding that when global histone hyperacetylation is induced by treatment with TSA, a substantial fraction [but not all] of the maturing oocytes become euploid (∼40%) [48]. Thus, even when the maturation-associated deacetylation of histones is inhibited and histone acetylation is increased, oocytes/eggs apparently have robust mechanisms to ensure proper chromosome segregation. This ability is consistent with the centrality of oocytes/eggs in reproduction, i.e., robust mechanisms to segregate properly chromosomes are an outcome of strong selective pressures to maintain reproductive fitness. The lower incidence of aneuploidy exhibited in eggs lacking HDAC2, when compared to TSA-treated oocytes, is consistent with mutant oocytes undergoing normal histone deacetylation, except for H4K16.

Acetylation of H4K16 inhibits formation of the higher order 30 nm chromatin structure, and loss of H4K16 shows defects equivalent to the loss of the H4 tails [49]. The increased acetylation of H4K16 in oocytes lacking HDAC2 is likely contributes to the failure of chromosomes to condense fully during oocyte maturation, despite deacetylation of other acetylated lysines. In addition, the increased incidence in mutant oocytes of kinetochores not able to interact with microtubules to form cold-stable microtubules is a functional assay confirming that kinetochore function is indeed compromised. The basis for compromised kinetochore function may reside in histone hyperacetylation interfering with proper kinetochore assembly [50] that leads to the observed decrease in CENP-A staining in mutant oocytes.

Although an age-dependent loss of cohesion can account for the majority of aneuploidies associated with increased maternal age [51], compromised histone deacetylation during maturation may be another source. For example, following maturation of mouse oocytes obtained from old mice, normal deacetylation of H4K8 and H4K12 do not occur, whereas H4K16 and H3K14 are properly deacetylated [48]. Likewise, less deacetylation of histone H4K12 occurs following maturation of oocytes obtained from older women when compared to younger women, and moreover, residual acetylation is correlated with misaligned chromosomes [52]. Thus, compromised histone deacetylation following oocyte maturation in general may result in an increased incidence of aneuploidy by leading to misaligned chromosomes on the meiotic spindle.

In summary, the results reported here provide further evidence for a critical role of HDAC2 in oocyte development and provide explanations for the infertility observed in *Hdac1^−/+^/Hdac2^−/−^* and sub-fertility in *Hdac2* ^−/−^ female mice. In particular, the results suggest that HDAC2 is largely responsible for deacetylation of H4K16 that occurs during oocyte maturation and that such deacetylation is critical for proper chromosome segregation.

## Materials and Methods

### Mouse Mutants and Collection of 1-Cell Embryos

Details for generating mutant mouse lines, histological analysis of ovaries, oocytes and embryos collection, RNA extraction and real time RT-PCR, immunostaining and immunoblot analysis are described in [14]. *Hdac1* or *Hdac2* mutants, in which the gene has only been deleted in oocytes, are referred to as *Hdac1* ^−/−^ or *Hdac2* ^−/−^, respectively. *Hdac1*-*Hdac2* mutants (double mutant) are referred to as *Hdac1:2* ^−/−^. *Hdac1* heterozygotes-*Hdac2* null oocytes are referred to as *Hdac1* ^−/+^/*Hdac2* ^−/−^ and *Hdac1* null-*Hdac2* heterozygote oocytes are referred to as *Hdac1* ^−/−^/*Hdac2* ^−/+^. To obtain 1-cell embryos for BrdU incorporation assays, *Hdac1* ^−/+^/*Hdac2* ^−/−^ female mice were superovulated with the injection of 5 IU of PMSG, followed 48 hours later by 5 IU of human chorionic gonadotropin (hCG). The mice were then mated with B6D2F1/J males (Jackson Laboratory, Bar Harbor, ME) and 1-cell embryos collected 24 h post hCG administration. Cumulus cells were removed by a brief hyaluronidase treatment (3 mg/ml). All animal experiments were approved by the institutional animal use and care committee and were consistent with the National Institutes of Health (NIH) guidelines.

### cRNA Preparation and Microinjection

Mouse *Hdac2* cDNA was cloned in a PIVT plasmid using standard recombinant DNA techniques. To prepare cRNAs, plasmids were linearized, and capped mRNAs were generated by *in vitro* transcription using T7 mMESSAGE mMachine (Ambion) according to the manufacturer's instructions. Following *in vitro* transcription, cRNA was polyadenylated using the PolyA Tailing Kit (Ambion). Synthesized cRNA was then purified using an MEGAclear Kit (Ambion), redissolved in RNase-free water, and stored at −80°C.

Full-grown oocytes were collected from mice of different genotypes and cultured in CZB [53] medium containing 0.2 mM IBMX in an atmosphere of 5% CO_2_ in air at 37°C. Microinjection of oocytes was performed as previously described [54]. Injections were done in 10-µl drops of modified Whitten's medium [55] containing 15 mM HEPES, pH 7.2, 7 mM Na_2_HCO_3_, 10 µg/ml gentamicin and 0.01% PVA containing 2.5 µM milrinone. Approximately 10 pl of *EGfp* or *Hdac2* cRNA was injected into the cytoplasm of GV oocytes using a PLI-100 Pico-Injector (Harvard Apparatus, Holliston, MA) on the stage of a Nikon TE2000 microscope equipped with Hoffman optics and Narishige micromanipulators. Following microinjection, oocytes were cultured in CZB plus IBMX for 24 h, and then the injected oocytes were matured by washing and culturing them in IBMX-free CZB medium for 18 h.

### In Vitro Transcription Assay

5-Ethynyl uridine (5EU) incorporation assays to detect transcription were performed as previously described [56]. Meiotically incompetent growing oocytes (or embryos) were cultured in the presence of 1 mM 5EU in CZB medium for oocytes or KSOM+AA for embryos [57] for 1 h and then fixed in 2% paraformaldehyde in PBS for 20 min at room temperature. 5EU incorporation into RNA was detected using Click-iT RNA Alexa Fluor 488 HCS Assay (Invitrogen) according to the manufacturer's protocol. Fluorescence was detected on a Leica TCS SP laser-scanning confocal microscope. The intensity of fluorescence was quantified using Image J software (National institutes of Health) as previously described [58].

### Incorporation of bromodeoxyuridine (BrdU)

Assays were conducted as previously described [32] except the 1-cell embryos were cultured for 5–8 h rather than 30 min in KSOM medium supplemented with 10 µM BrdU (Sigma).

### Monastrol Treatment, Kinetochore Immunocytochemistry, and Chromosome Counting

Monastrol treatment, immunocytochemical detection of kinetochores and chromosome counting were performed as previously described [51]. Images were collected with a spinning disk confocal miroscope at 0.4 µm intervals to span the entire region of the spindle, using a 100×1.4 NA oil immersion objective. To obtain a chromosome count for each egg, serial confocal sections were analyzed to determine the total number of kinetochores.

### Cold-Stable Microtubule Assay

Wild-type, *Hdac2^−/−^* and *Hdac1^−/+^/Hdac2^−/−^* full-grown oocytes were matured in CZB medium for 7 h to MI and then transferred to in MEM medium and placed on ice for 8 min before fixing in 2% paraformaldehyde for 20 min. Immunocytochemistry was performed with CREST autoimmune serum and anti-TUBB antibody to label kinetochores and microtubules, respectively. Images were collected with a spinning disk confocal miroscope at 0.4 µm intervals to span the entire region of the spindle, using a 100×1.4 NA oil immersion objective as described before [51].

### TUNEL Labeling Assay

TUNEL (TdT-mediated dUTP nick end labeling) assays were performed with an In Situ Cell Death Detection Kit (Roche Diagnostics, Basel, Switzerland) according to the manufacturer's instructions.

### Antibodies

The following antibodies were used for immunofluorescence and/or immunoblotting blotting: anti-CDC6 rabbit polyclonal antibody (11640-1AP; Proteintech; IF, 1∶200), anti-P21 mouse monoclonal antibody (556430, BD Pharmingen; IF, 1∶100), anti-P57 rabbit monoclonal antibody (2372-1; Epitomics; IF, 1∶200), anti-ORC6L rat monoclonal antibody (4737; Cell signaling; IF, 1∶100), and anti-CENP-A rabbit monoclonal antibody (2048; Cell signaling; IF, 1∶200). All the other antibodies used in this paper have been described previously [14].

### Statistics

Experiments were performed at least three times and the values are presented as mean ± SEM. All proportional data were subjected to an arcsine transformation before statistical analysis. Statistics were calculated with Microsoft Excel software. A P-value of <0.05 was considered to be statistically significant.

## Supporting Information

Figure S1 Depletion of HDAC1 in oocytes has little effect on oocyte maturation and preimplantation development. (A) Relative abundance of *Hdac1* and *Hdac2* transcripts in full-grown oocytes obtained from WT and *Hdac1^−/−^* mice. Data are expressed relative to that in WT oocytes. The experiment was performed four times and the data expressed as mean ± SEM. *, p<0.05. (B) Total protein was extracted from 300 growing or full-grown oocytes obtained from WT, *Hdac1^−/−^*, and *Hdac2^−/−^* mice for immunoblotting. The experiment was performed two times and similar results were obtained for each experiment. Note that the amount of HDAC1 protein displays a decrease during oocyte growth. Thus, the decrease in the relative amount of HDAC1 in incompetent oocytes appears greater than in full-grown oocytes. (C) Similar numbers of ovulated eggs are obtained from WT and *Hdac1^−/−^* mice after hormonal stimulation. At least 6 mice of each genotype were used, and the average number of ovulated oocytes per female is indicated. (D) Tubulin immunofluorescence staining of WT and *Hdac1^−/−^* MII eggs exhibiting normal spindles in both oocytes (upper panel). DNA was counterstained with sytox green. The bar corresponds to 10 µm. One-cell embryos were collected from WT and *Hdac1^−/−^* mice and cultured 96 h in KSOM; representative bright-field photographs were showed in lower panel. The experiment was conducted 3 times. At least 100 embryos from each genotype were analyzed. The bar corresponds to 80 µm.
(TIF)

Figure S2 Effects of HDAC2 depletion in oocytes on HDAC1 and HDAC2 expression and preimplantation development. (A) Representative bright-field photographs from an embryo culture experiment. Maternally depleted HDAC2 and WT 1-cell embryos were collected 20 h after HCG injection then cultured for 24 h, 48 h, 72 h and 96 h. The bar corresponds to 80 µm. (B) Different stages of oocytes and embryos were collected from HDAC2 depleted and WT mice and were processed for immunocytochemical detection of HDAC1 and HDAC 2. Strong nuclear staining of HDAC1 was found in HDAC2 depleted GV oocytes, 1-cell and 2-cell embryos. At least 20 oocytes/embryos from each genotype at the indicated times were analyzed, and the experiment was conducted 3 times. Shown are representative images. The bar corresponds to 40 µm.
(TIF)

Figure S3 Deletion of maternal HDAC2 has little effect on other histone lysine actylation following oocyte maturation. Immunocytochemical detection of histone H3K4, H3K9 and H3K14 acetylation (A) or histone H4K5, H4K8 and H4K12 acetylation (B) in WT, *Hdac2^−/−^* and *Hdac1^−/+^/Hdac2* ^−/−^ MII eggs; at least 20 oocytes from each genotype were analyzed, and the experiment was conducted 2 times. Shown are representative images and the DNA was stained with Sytox Green. The bar corresponds to 10 µm.
(TIF)

Figure S4 Depletion of HDAC2 has no effect on the expression of MYST1 and the acetylation state of H4K16 in full-grown oocytes. (A) Immunocytochemical detection of MYST1 in WT, *Hdac2^−/−^* and *Hdac1^−/+^/Hdac2* ^−/−^ full-grown oocytes; at least 20 oocytes from each genotype were analyzed, and the experiment was conducted 3 times. Shown are representative images and the DNA was stained with Sytox Green. The bar corresponds to 10 µm. (B) Immunocytochemical detection of H4K16 acetylation in WT and *Hdac1^−/+^/Hdac2* ^−/−^ full-grown oocytes; at least 20 oocytes from each genotype were analyzed, and the experiment was conducted 3 times. Shown are representative images and the HDAC2 was also detected. The bar corresponds to 10 µm.
(TIF)

Figure S5 Deletion of maternal HDAC2 causes aneuploidy in mouse eggs. MII eggs from WT, *Hdac2^−/−^* and *Hdac1^−/+^/Hdac2^−/−^* hormone-primed mice were treated with monastrol to disperse the chromosomes, and then fixed and stained for DNA (Sytox, green) and kinetochores (CREST, red). Shown are representative images. The bar corresponds to 5 µm. WT egg is euploid with 20 paired sister kinetochores (numbered 1–40), whereas, the final kinetochore count in *Hdac2^−/−^* and *Hdac1^−/+^/Hdac2^−/−^* eggs is 38 and 37 respectively.
(TIF)

Figure S6 Deletion of maternal HDAC2 results in 1-cell embryos block in G1 phase independent of P57 and P21. (A) Up-regulation of CDK inhibitors p21^WAF1/CIP1^ and p57^Kip2^ in *Hdac1^−/+^/Hdac2* ^−/−^ 1-cell embryos. The relative abundance of G1 phase specific CDK inhibitors transcripts was assayed by qRT-PCR and expressed relative to their WT mRNA levels. UBF was used as internal control. The experiment was performed 3 times and the data expressed as mean ± SEM. *, p<0.05. (B) Immunocytochemical detection of P57 and P21 in WT and *Hdac1^−/+^/Hdac2* ^−/−^ 1-cell embryos. At least 20 embryos from each genotype were analyzed, and the experiment was conducted 2 times. Shown are representative images. The bar corresponds to 35 µm. (C) Immunocytochemical detection of ORC6L and CDC6 in WT and *Hdac1^−/+^/Hdac2* ^−/−^ 1-cell embryos. At least 20 embryos from each genotype were analyzed, and the experiment was conducted 2 times. Shown are representative images. The bar corresponds to 35 µm.
(TIF)

Figure S7 Depletion of HDAC2 has little effect on histone modifications in NSN or SN full-grown oocytes. Immunocytochemical detection of histone acetylated H4K5, H4K8, H4K12 and H4K16 (A) or histone H3K4me1, H3K4me2 and H3K4me3 (B) in NSN or SN WT or *Hdac2* ^−/−^ oocytes. At least 20 oocytes from each genotype were analyzed, and the experiment was conducted 2 times. Shown are representative images and the DNA was stained with Sytox Green (green). The bar corresponds to 10 µm. (C) Immunocytochemical detection of histone H4K16 acetylation in WT, *Hdac1^−/−^* and *Hdac1^−/−^/Hdac2* ^−/+^ MII eggs; at least 20 oocytes from each genotype were analyzed, and the experiment was conducted 2 times. Shown are representative images and the DNA was stained with Sytox Green (green). The bar corresponds to 10 µm. Because acetylated histone H4K16 is extensively deacetylated during maturation, the laser power was increased so that the signal could readily be detected.
(TIF)
