# Supplementary figures and images for: Histone Deacetylase 2 (HDAC2) Regulates Chromosome Segregation and Kinetochore Function via H4K16 Deacetylation during Oocyte Maturation in Mouse
Source: PLoS Genet. 2013 Mar 14;9(3):e1003377. doi: 10.1371/journal.pgen.1003377 (PMC3597510; doi:10.1371/journal.pgen.1003377)

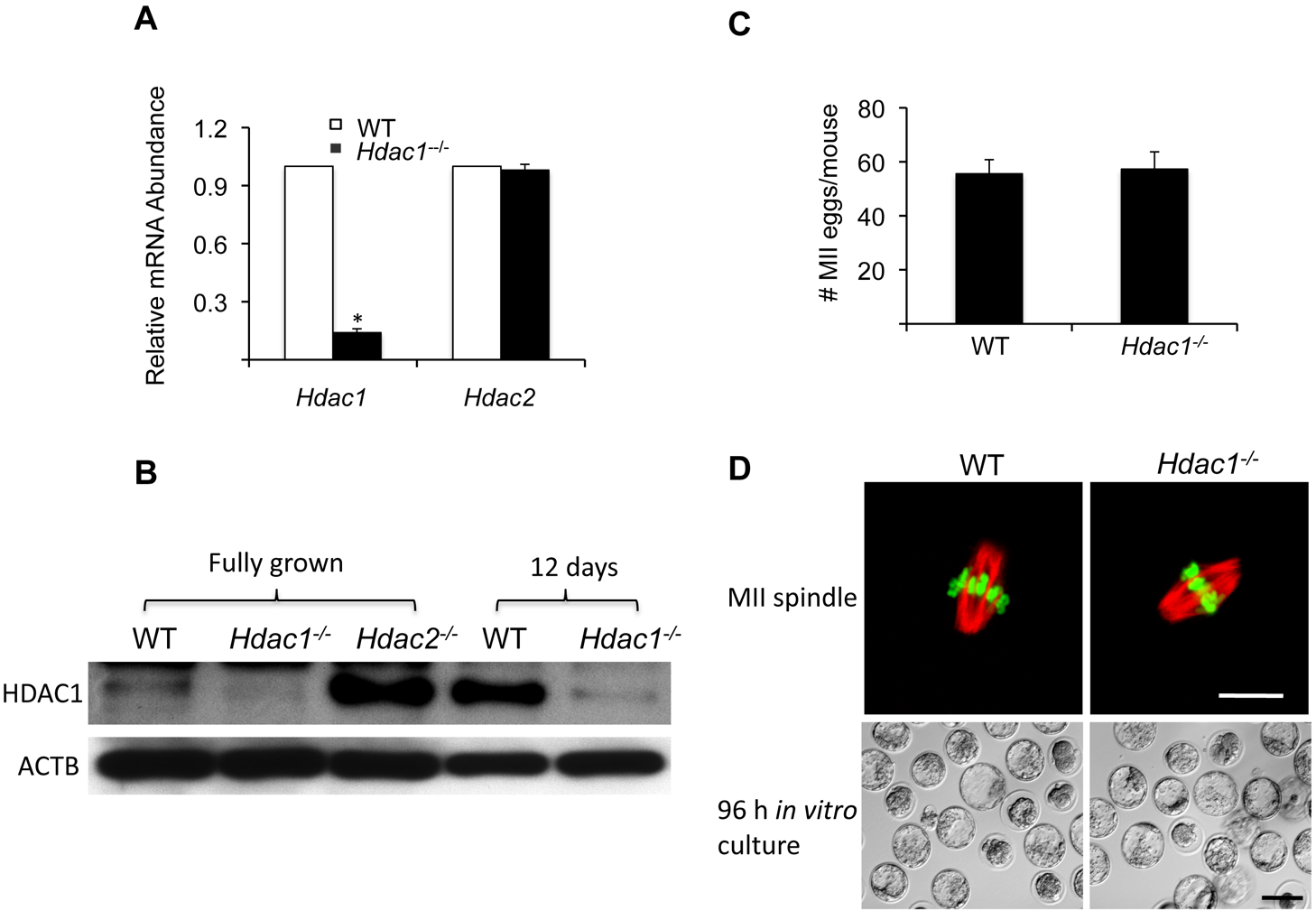

Supplement: Figure S1 — Depletion of HDAC1 in oocytes has little effect on oocyte maturation and preimplantation development. (A) Relative abundance of Hdac1 and Hdac2 transcripts in full-grown oocytes obtained from WT and Hdac1−/− mice. Data are expressed relative to that in WT oocytes. The experiment was performed four times and the data expressed as mean ± SEM. *, p<0.05. (B) Total protein was extracted from 300 growing or full-grown oocytes obtained from WT, Hdac1−/−, and Hdac2−/− mice for immunoblotting. The experiment was performed two times and similar results were obtained for each experiment. Note that the amount of HDAC1 protein displays a decrease during oocyte growth. Thus, the decrease in the relative amount of HDAC1 in incompetent oocytes appears greater than in full-grown oocytes. (C) Similar numbers of ovulated eggs are obtained from WT and Hdac1−/− mice after hormonal stimulation. At least 6 mice of each genotype were used, and the average number of ovulated oocytes per female is indicated. (D) Tubulin immunofluorescence staining of WT and Hdac1−/− MII eggs exhibiting normal spindles in both oocytes (upper panel). DNA was counterstained with sytox green. The bar corresponds to 10 µm. One-cell embryos were collected from WT and Hdac1−/− mice and cultured 96 h in KSOM; representative bright-field photographs were showed in lower panel. The experiment was conducted 3 times. At least 100 embryos from each genotype were analyzed. The bar corresponds to 80 µm. (TIF) [file pgen.1003377.s001.tif]

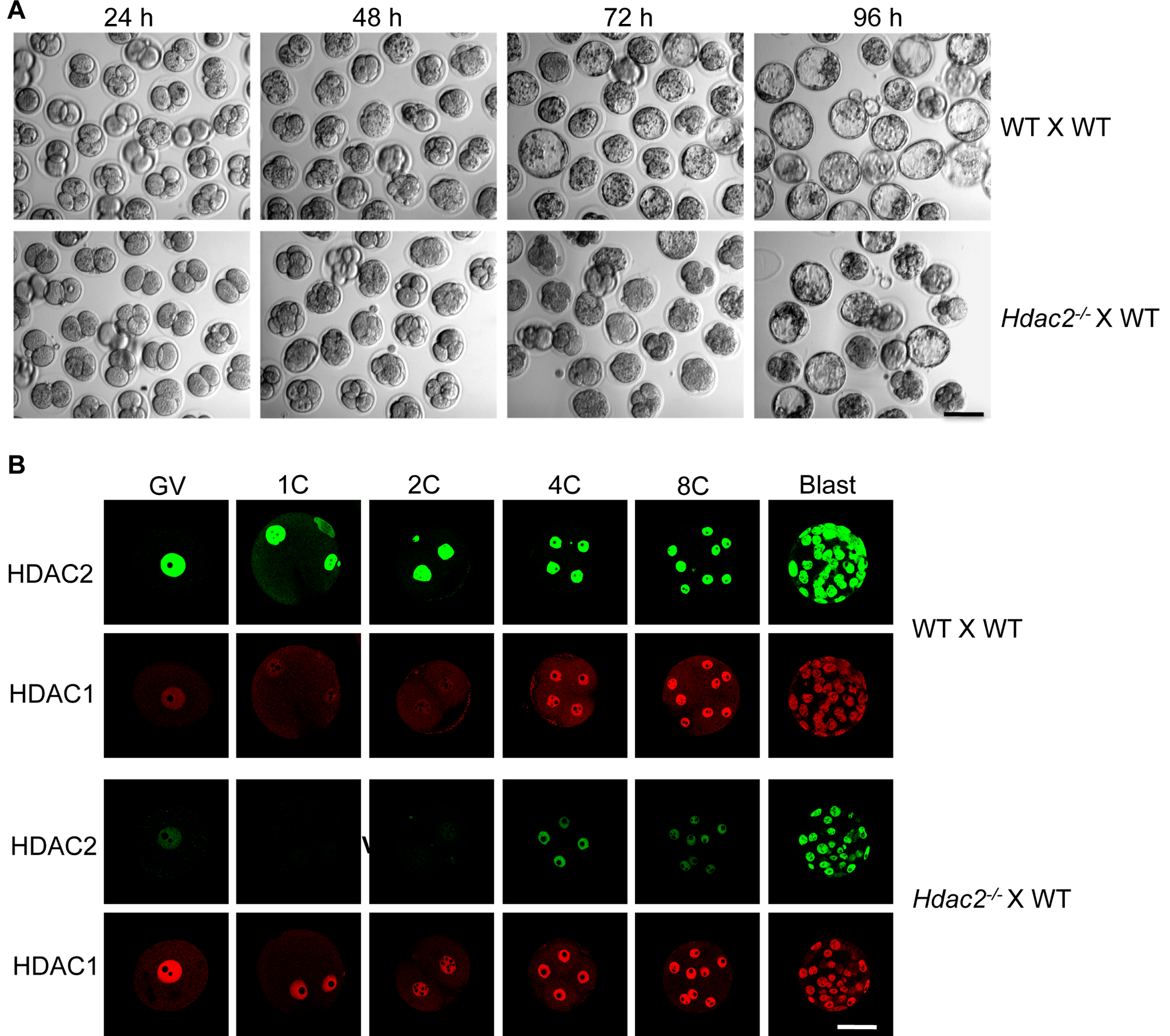

Supplement: Figure S2 — Effects of HDAC2 depletion in oocytes on HDAC1 and HDAC2 expression and preimplantation development. (A) Representative bright-field photographs from an embryo culture experiment. Maternally depleted HDAC2 and WT 1-cell embryos were collected 20 h after HCG injection then cultured for 24 h, 48 h, 72 h and 96 h. The bar corresponds to 80 µm. (B) Different stages of oocytes and embryos were collected from HDAC2 depleted and WT mice and were processed for immunocytochemical detection of HDAC1 and HDAC 2. Strong nuclear staining of HDAC1 was found in HDAC2 depleted GV oocytes, 1-cell and 2-cell embryos. At least 20 oocytes/embryos from each genotype at the indicated times were analyzed, and the experiment was conducted 3 times. Shown are representative images. The bar corresponds to 40 µm. (TIF) [file pgen.1003377.s002.tif]

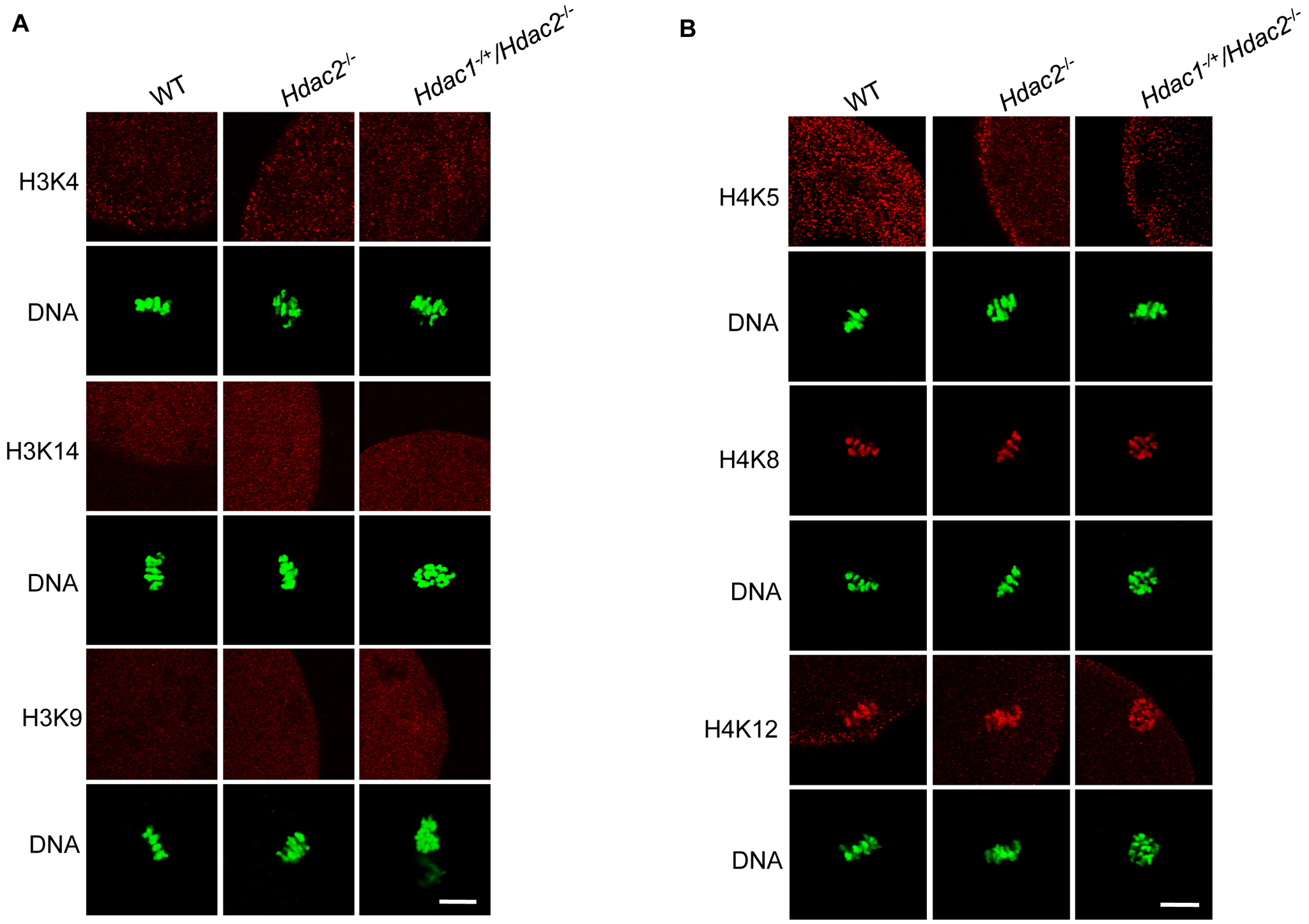

Supplement: Figure S3 — Deletion of maternal HDAC2 has little effect on other histone lysine actylation following oocyte maturation. Immunocytochemical detection of histone H3K4, H3K9 and H3K14 acetylation (A) or histone H4K5, H4K8 and H4K12 acetylation (B) in WT, Hdac2−/− and Hdac1−/+/Hdac2 −/− MII eggs; at least 20 oocytes from each genotype were analyzed, and the experiment was conducted 2 times. Shown are representative images and the DNA was stained with Sytox Green. The bar corresponds to 10 µm. (TIF) [file pgen.1003377.s003.tif]

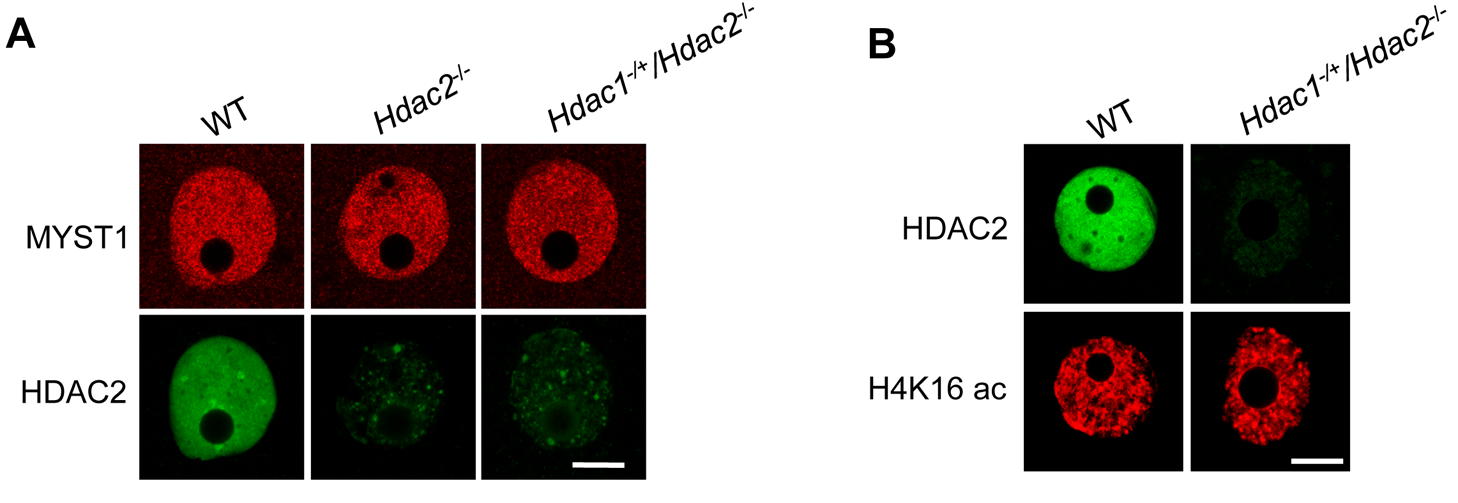

Supplement: Figure S4 — Depletion of HDAC2 has no effect on the expression of MYST1 and the acetylation state of H4K16 in full-grown oocytes. (A) Immunocytochemical detection of MYST1 in WT, Hdac2−/− and Hdac1−/+/Hdac2 −/− full-grown oocytes; at least 20 oocytes from each genotype were analyzed, and the experiment was conducted 3 times. Shown are representative images and the DNA was stained with Sytox Green. The bar corresponds to 10 µm. (B) Immunocytochemical detection of H4K16 acetylation in WT and Hdac1−/+/Hdac2 −/− full-grown oocytes; at least 20 oocytes from each genotype were analyzed, and the experiment was conducted 3 times. Shown are representative images and the HDAC2 was also detected. The bar corresponds to 10 µm. (TIF) [file pgen.1003377.s004.tif]

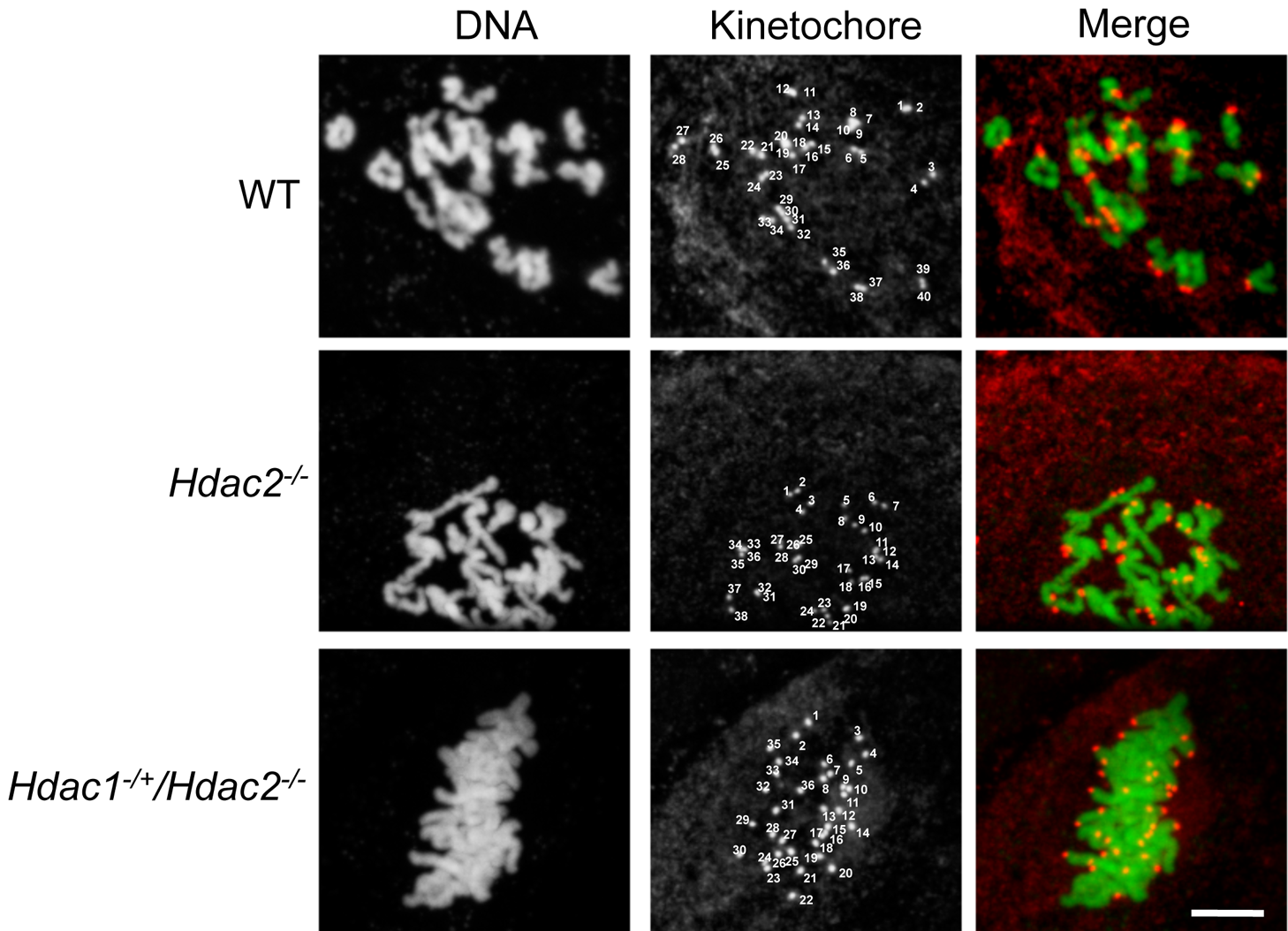

Supplement: Figure S5 — Deletion of maternal HDAC2 causes aneuploidy in mouse eggs. MII eggs from WT, Hdac2−/− and Hdac1−/+/Hdac2−/− hormone-primed mice were treated with monastrol to disperse the chromosomes, and then fixed and stained for DNA (Sytox, green) and kinetochores (CREST, red). Shown are representative images. The bar corresponds to 5 µm. WT egg is euploid with 20 paired sister kinetochores (numbered 1–40), whereas, the final kinetochore count in Hdac2−/− and Hdac1−/+/Hdac2−/− eggs is 38 and 37 respectively. (TIF) [file pgen.1003377.s005.tif]

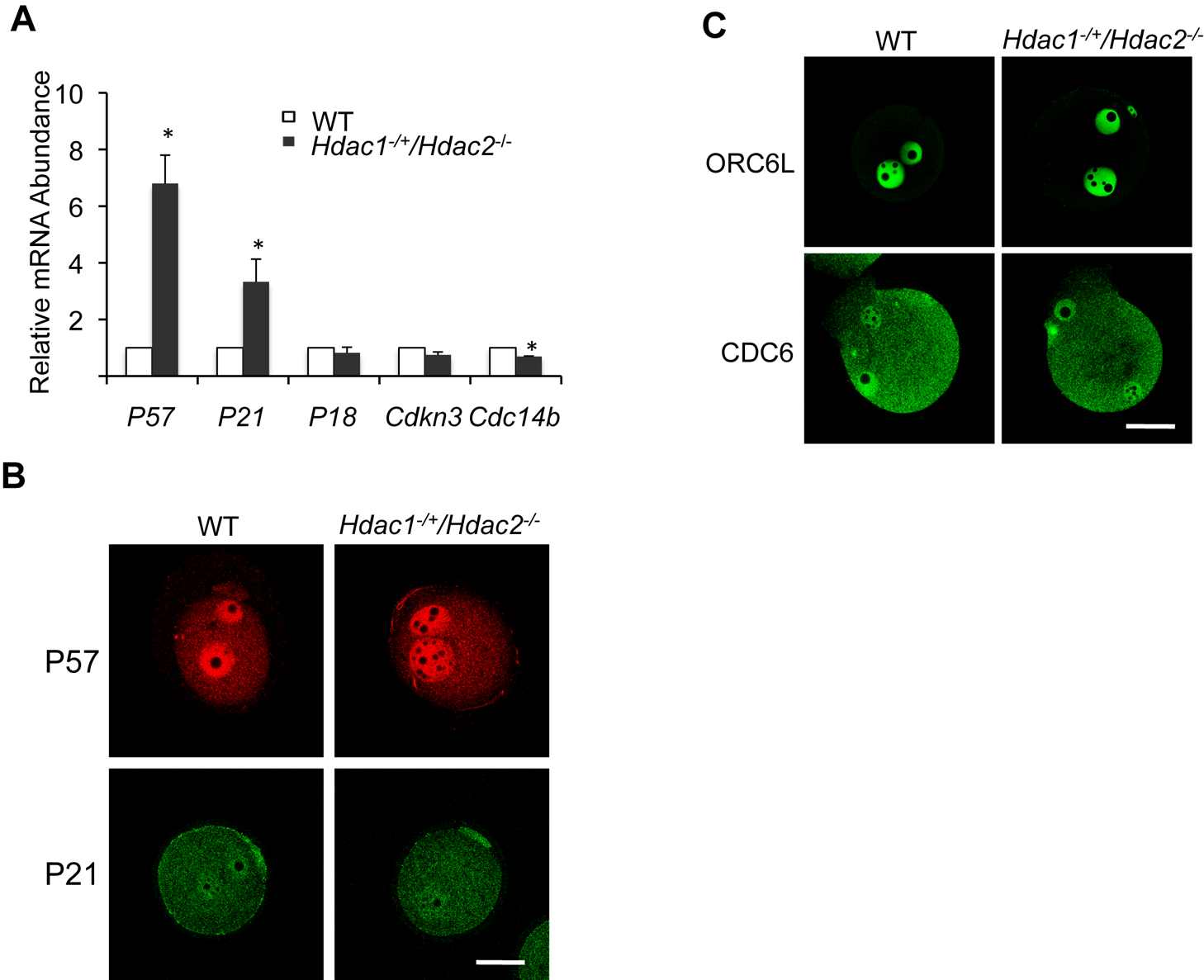

Supplement: Figure S6 — Deletion of maternal HDAC2 results in 1-cell embryos block in G1 phase independent of P57 and P21. (A) Up-regulation of CDK inhibitors p21WAF1/CIP1 and p57Kip2 in Hdac1−/+/Hdac2 −/− 1-cell embryos. The relative abundance of G1 phase specific CDK inhibitors transcripts was assayed by qRT-PCR and expressed relative to their WT mRNA levels. UBF was used as internal control. The experiment was performed 3 times and the data expressed as mean ± SEM. *, p<0.05. (B) Immunocytochemical detection of P57 and P21 in WT and Hdac1−/+/Hdac2 −/− 1-cell embryos. At least 20 embryos from each genotype were analyzed, and the experiment was conducted 2 times. Shown are representative images. The bar corresponds to 35 µm. (C) Immunocytochemical detection of ORC6L and CDC6 in WT and Hdac1−/+/Hdac2 −/− 1-cell embryos. At least 20 embryos from each genotype were analyzed, and the experiment was conducted 2 times. Shown are representative images. The bar corresponds to 35 µm. (TIF) [file pgen.1003377.s006.tif]

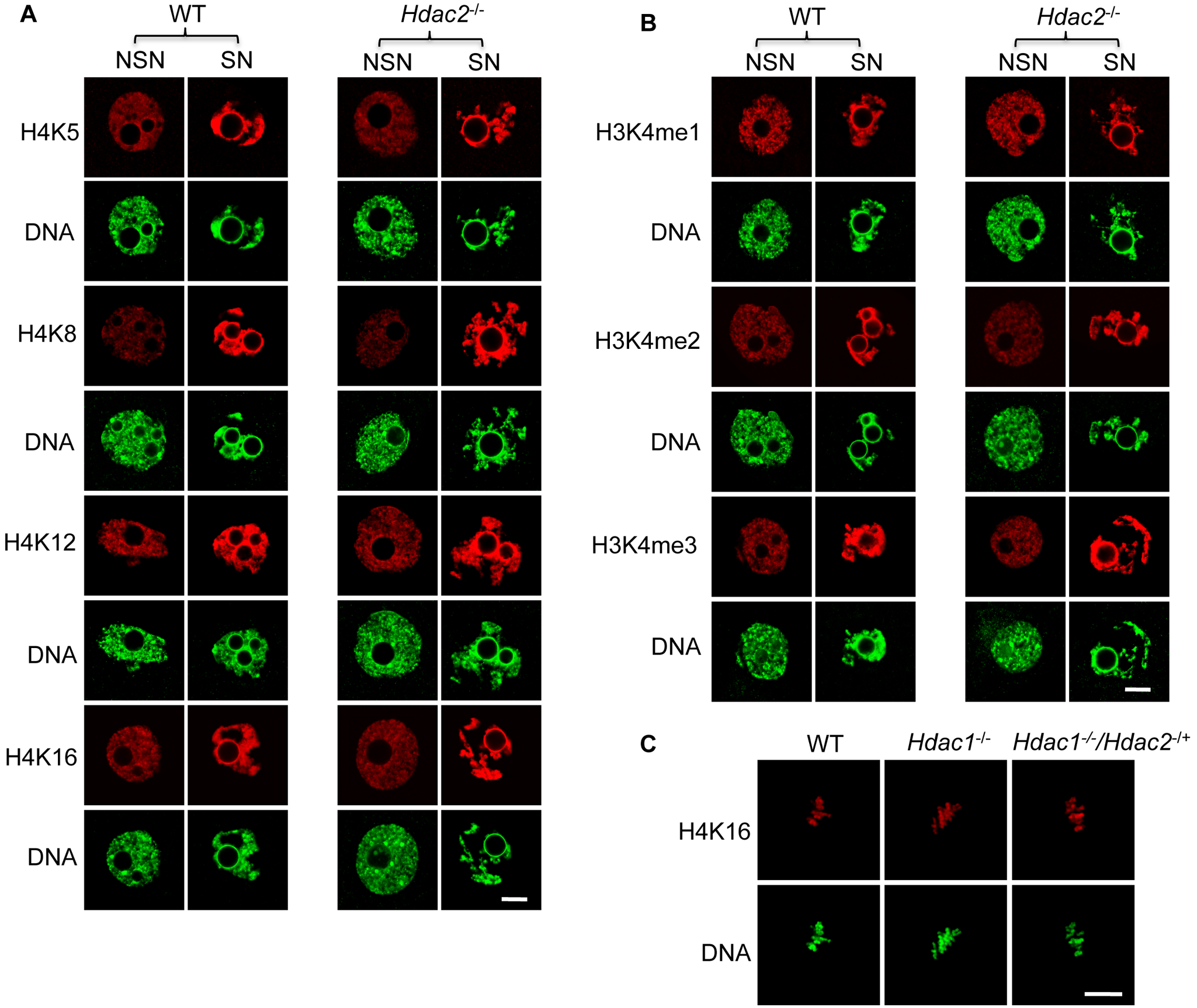

Supplement: Figure S7 — Depletion of HDAC2 has little effect on histone modifications in NSN or SN full-grown oocytes. Immunocytochemical detection of histone acetylated H4K5, H4K8, H4K12 and H4K16 (A) or histone H3K4me1, H3K4me2 and H3K4me3 (B) in NSN or SN WT or Hdac2 −/− oocytes. At least 20 oocytes from each genotype were analyzed, and the experiment was conducted 2 times. Shown are representative images and the DNA was stained with Sytox Green (green). The bar corresponds to 10 µm. (C) Immunocytochemical detection of histone H4K16 acetylation in WT, Hdac1−/− and Hdac1−/−/Hdac2 −/+ MII eggs; at least 20 oocytes from each genotype were analyzed, and the experiment was conducted 2 times. Shown are representative images and the DNA was stained with Sytox Green (green). The bar corresponds to 10 µm. Because acetylated histone H4K16 is extensively deacetylated during maturation, the laser power was increased so that the signal could readily be detected. (TIF) [file pgen.1003377.s007.tif]
